# Novel monoclonal antibodies to study tissue regeneration in planarians

**DOI:** 10.1186/s12861-014-0050-9

**Published:** 2015-01-21

**Authors:** Kelly G Ross, Kerilyn C Omuro, Matthew R Taylor, Roma K Munday, Amy Hubert, Ryan S King, Ricardo M Zayas

**Affiliations:** Department of Biology, San Diego State University, San Diego, CA 92182 USA; Howard Hughes Medical Institute, Department of Cell and Developmental Biology, University of Illinois at Urbana-Champaign, 601 S. Goodwin Ave., Urbana, IL 61801 USA; Present address: Department of Biological Sciences, Southern Illinois University Edwardsville, Edwardsville, IL 62026 USA; Present address: Department of Biology, St. Norbert College, De Pere, WI 54115 USA

**Keywords:** Planaria, Regeneration, *Schmidtea mediterranea*, Monoclonal antibodies, Immunostaining, Immunohistochemistry

## Abstract

**Background:**

Planarians are an attractive model organism for studying stem cell-based regeneration due to their ability to replace all of their tissues from a population of adult stem cells. The molecular toolkit for planarian studies currently includes the ability to study gene function using RNA interference (RNAi) and observe gene expression via *in situ* hybridizations. However, there are few antibodies available to visualize protein expression, which would greatly enhance analysis of RNAi experiments as well as allow further characterization of planarian cell populations using immunocytochemistry and other immunological techniques. Thus, additional, easy-to-use, and widely available monoclonal antibodies would be advantageous to study regeneration in planarians.

**Results:**

We have created seven monoclonal antibodies by inoculating mice with formaldehyde-fixed cells isolated from dissociated 3-day regeneration blastemas. These monoclonal antibodies can be used to label muscle fibers, axonal projections in the central and peripheral nervous systems, two populations of intestinal cells, ciliated cells, a subset of neoblast progeny, and discrete cells within the central nervous system as well as the regeneration blastema. We have tested these antibodies using eight variations of a formaldehyde-based fixation protocol and determined reliable protocols for immunolabeling whole planarians with each antibody. We found that labeling efficiency for each antibody varies greatly depending on the addition or removal of tissue processing steps that are used for *in situ* hybridization or immunolabeling techniques. Our experiments show that a subset of the antibodies can be used alongside markers commonly used in planarian research, including anti-SYNAPSIN and anti-SMEDWI, or following whole-mount *in situ* hybridization experiments.

**Conclusions:**

The monoclonal antibodies described in this paper will be a valuable resource for planarian research. These antibodies have the potential to be used to better understand planarian biology and to characterize phenotypes following RNAi experiments. In addition, we present alterations to fixation protocols and demonstrate how these changes can increase the labeling efficiencies of antibodies used to stain whole planarians.

**Electronic supplementary material:**

The online version of this article (doi:10.1186/s12861-014-0050-9) contains supplementary material, which is available to authorized users.

## Background

Planarians, free-living flatworms with an extraordinary ability to regenerate, are regarded as an excellent model system for regenerative studies. These animals possess the ability to regenerate an entire organism from small body fragments from a population of adult stem cells (neoblasts) [1-3]. Recently, single neoblast transplantation experiments demonstrated that a subset of these cells (clonogenic neoblasts) are truly pluripotent and can differentiate into any planarian cell type or tissue lost by amputation, injury, or normal physiological turnover [4].

**Figure 1:**
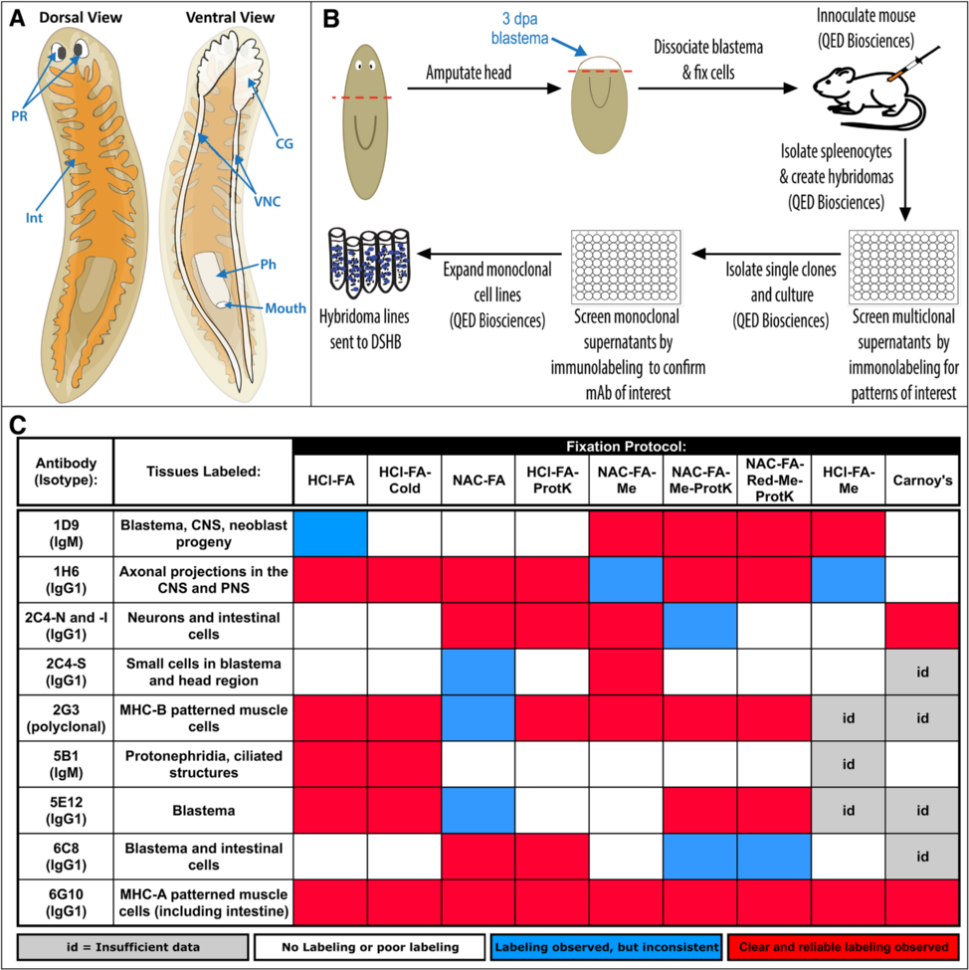
**Overview of the basic anatomy of asexual** ***Schmidtea mediterranea*** **recognized by the monoclonal antibodies generated in this study and tissue fixation protocols tested. (A)** Cartoon of the basic anatomy of asexual *S. mediterranea* with arrows highlighting some of the major organs labeled with the monoclonal antibodies generated in this study. PR, photoreceptors; Int, intestine; CG, cephalic ganglia; VNC, ventral nerve cords; Ph, pharynx. **(B)** Summary of the creation of the monoclonal antibodies used in the subsequent experiments. dpa: days post amputation. **(C)** A heat map summarizing the labeling efficiency for each antibody following eight variations of a formaldehyde-based fixation protocol or Carnoy’s fixation. For each fixation and antibody, at least 2 experiments were performed with ≥ 4 worms, which were scored independently by 2 or more individuals. The fixation protocols are named according to the reagents used for each processing step. HCl, hydrochloric acid; FA, formaldehyde; ProtK, Proteinase-K; NAC, N-Acetyl Cysteine; Me, methanol; Red, reduction solution.

Planarians have several distinct major organ systems (illustrated in Figure 1A). They possess a centralized nervous system (CNS), consisting of bi-lobed cephalic ganglia, a brain-like structure located at the anterior end of the animal, connected to two longitudinal ventral nerve cords (VNC) that project posteriorly along the length of the worm [5-7]. The majority of planarians’ light detection is achieved by their photoreceptors, which are rich in photosensitive pigment cells and photoreceptor neurons that are located on the anterior dorsal surface of the head [8]. Planarians possess a blind digestive system (also referred to as the gastrovascular system) that consists of a pharynx, through which they ingest food and defecate, connected to three primary intestinal branches that distribute nutrients [9-12]. They excrete soluble waste and maintain osmoregularity with their protonephridial systems, tubular structures that extend to the exterior of the animals and are analogous to the vertebrate kidney [13-15]. Thus, we can use planarians to study how defined organ systems are regenerated from adult stem cells.

There have been many great advances in the past decade in identifying and optimizing tools to study the molecular basis of planarian regeneration. Gene expression can be inhibited using RNA interference (RNAi), which allows the study of gene function [16]. Genomic sequencing of *Schmidtea mediterranea* and the availability of multiple transcriptomes combined with custom microarrays or mRNA sequencing have facilitated identification of genes involved in the regeneration of planarian organ systems (recently reviewed in [17]). Whole-mount *in situ* hybridization protocols have been developed and optimized for the visualization of gene expression in planarians [16,18,19]; this information can be coupled with functional analyses to determine the role specific genes play in tissue regeneration. Further, fluorescent lectins have been utilized to label several cell types in planarians, including secretory cells and the reproductive organs of hermaphroditic strains [20,21]. However, there is a dearth of cell-type and tissue-specific antibodies to examine the effects of experimental manipulation in planarians. Available antibodies known to label tissues in *S. mediterranea* include a handful of antibodies created against well-conserved antigens in other species, such as anti-Phospho-Tyrosine (used in planarian studies to label the gut and central nervous system) [22,23], anti-Tubulin, which recognizes ciliated epithelium and neurons [24], and anti-Acetylated Tubulin can be used to visualize ciliated structures, including protonephridia [16,25]. Cebrià *et al.* [6] identified five antibodies (anti-SYNAPSIN, anti-5HT, anti-allatostatin, anti-GYRFamide, and anti-neuropeptide F) that cross-react with neurons in the CNS of *S. mediterranea* [6]. A small selection of monoclonal and polyclonal antibodies have been created against *S. mediterranea* antigens such as anti-SMEDWI, which labels planarian stem cells and their progeny [23]. TMUS-13, originally generated against *Dugesia tigrina* [26], has since been used to label the musculature in *S. mediterranea* [16], and monoclonal antibodies that recognize plasma membrane proteins on subsets of cells within X-ray sensitive and insensitive populations have also been created [27].

Additional antibodies will be useful to further characterize the cellular diversity found within planarian tissues, to track differentiation of planarian cell types, and to expand our understanding of the distribution and dynamics of tissue repair and replacement following wounding events. Discovery of cell surface markers would allow for sorting of specific cell populations, enabling the analysis of gene expression profiles for defined cell populations like the transcriptional profiles available for the heterogeneous irradiation sensitive populations, X1 (highly enriched for cycling neoblasts) and X2 (enriched for progenitor cells) [28,29]. Finally, it would be advantageous to have additional markers available for analyzing regeneration phenotypes following RNAi experiments.

Here, we report on the generation of monoclonal antibodies that recognize tissues in *S. mediterranea*. These antibodies were created by inoculating mice with formaldehyde-fixed cells derived from 3-day head blastemas. We tested the utility of these reagents for immunocytochemistry using multiple fixation protocols on intact and regenerating planarians and determined the optimal conditions for each antibody in asexual *S. mediterranea*. We describe this new set of markers and their staining patterns in muscle, neural structures, ciliated structures (including protonephridia), intestinal cells, and stem cell progeny. These antibodies are currently available to the community through the Developmental Studies Hybridoma Bank (DSHB).

## Results and discussion

To generate monoclonal antibodies (mAbs) that label planarian neoblast progeny and differentiated cell populations, we isolated cells from regeneration blastemas. Planarians were amputated pre-pharyngeally, and trunk fragments were allowed to initiate regeneration of a new head. At 3 days post-amputation (dpa), regeneration blastemas were isolated by transverse cutting, dissociated into single cells, fixed with formaldehyde, and used to inoculate mice to create hybridoma lines (see Methods and Figure 1B). We tested supernatants from 576 hybridoma lines by immunostaining intact and regenerating planarians; 236 supernatants were positive for staining in 3 dpa regeneration blastemas, discrete cell populations, or tissues in formaldehyde-fixed planarians. We selected 126 hybridomas for expansion and re-testing. The majority (80%) of these 126 hybridomas were positive for immunostaining in 3 dpa blastemas. Based on the staining patterns, signal and background intensities, 42 lines were chosen for additional expansion and re-screened in planarians, using eight different fixation protocols (Figure 1C; Additional file 1). When re-tested, 33 of 42 were positive, and 17 were selected for hybridoma sub-cloning. Seven monoclonal hybridoma cell-lines and one polyclonal antibody were successfully generated (Table 1). Below, we describe the labeling patterns of these antibodies.

**Table 1:** **Summary of monoclonal antibodies generated from selected hybridoma cell lines**

**Parental hybridoma**	**Clone at DSHB**	**Isotype**	**Dilution factor**	**Concentration of DSHB supernatant tested (μg/ml)**	**Tissues labeled**
1D9	E11	IgM kappa	500	34	Blastema, brain primordia, neoblast progeny
1H6	E9	IgG1 kappa	1000	54	Axonal projections in CNS and PNS
2C4	C2	IgG1 kappa	1000	26	Blastema, neurons, intestinal cells, anterior cells
2G3 (polyclonal)	N/A	N/A	Undiluted	N/A	Muscle fibers
5B1	E6	IgM kappa	1000	18	Protonephria, ciliated structures
5E12	E3	IgG1 kappa	1000	35	Blastema
6C8	A2	IgG1 kappa	1000	26	Blastema, intestinal cells
6G10	2C7	IgG1 kappa	1000	59	Muscle fibers

### Smed-6G10 and -2G3 label planarian musculature

**Figure 2:**
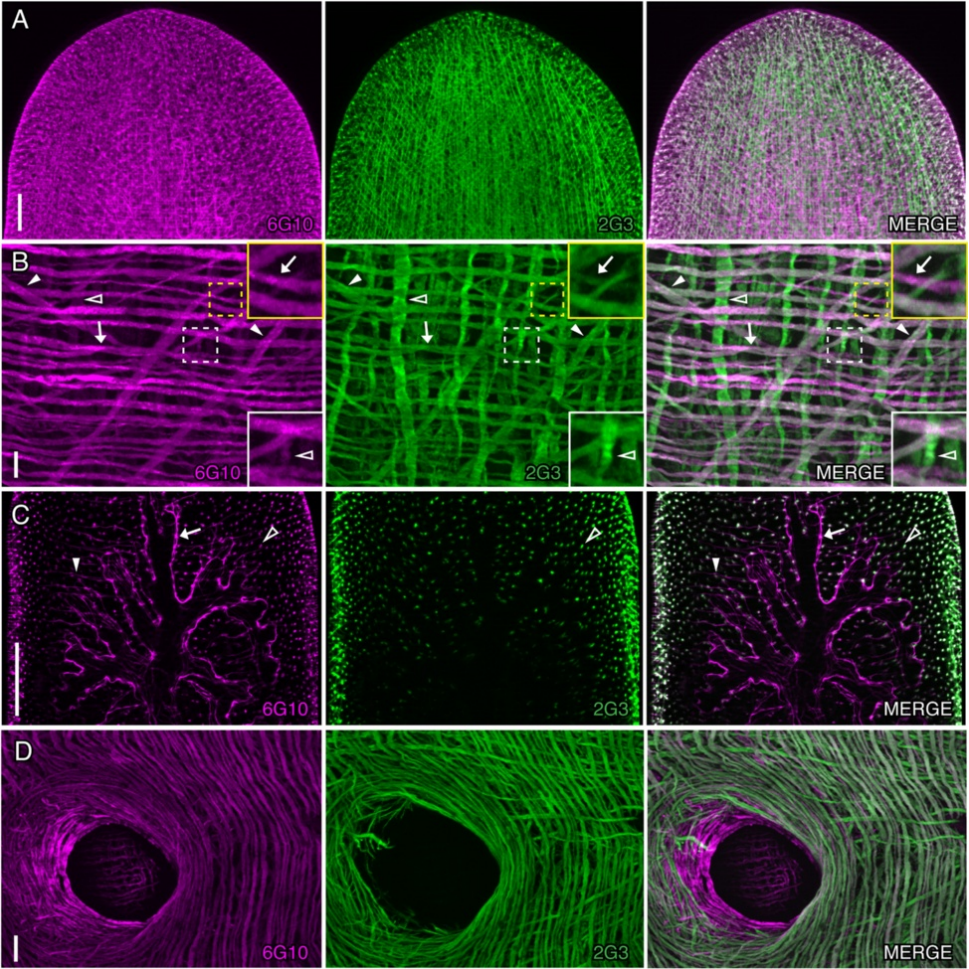
**Smed-6G10 and -2G3 label different populations of muscle fibers. (A-D)** Whole-mount immunostaining of intact planarians with 6G10 (magenta) and 2G3 (green). **(A)** 6G10 and 2G3 staining in the head region of the animal showing partial co-labeling. **(B)** 6G10 predominantly labels the circular and diagonal fibers, whereas 2G3 strongly labels circular, longitudinal, and diagonal fibers in the body wall musculature. Arrows indicate circular fibers. Closed arrowheads mark diagonal fibers. Open arrowheads highlight an example of a longitudinal fiber weakly labeled with 6G10. Insets show zoomed in regions marked by dashed white and yellow boxes. **(C)** Image of the anterior intestinal branches showing that 6G10 labels the intestinal musculature and transverse muscle fibers (marked by arrows and closed arrowheads, respectively). Open arrowhead highlights a transverse fiber in the parenchyma, which is co-labeled by 6G10 and 2G3. **(D)** 6G10 labels the circular muscle fibers of the body wall, mouth, and the pharynx; 2G3 labels circular, longitudinal, and diagonal fibers and the exterior portion of the mouth. Images are maximum intensity projections of optical sections. Anterior of the animal is to the top in **A**-**C**, and to the left in **D**. Scale bars: **(A, D)** 100 μm; **(B, D)** 10 μm.

Planarians have a subepidermal muscle wall that consists of four layers of muscle fibers: circular, longitudinal, diagonal, and a final layer of longitudinal fibers located between two nerve plexuses [10,30,31]. Additionally, there are abundant fibers traversing the parenchyma along the dorsoventral axis, muscle fibers surrounding the intestine, pharynx, and the mouth, and some transverse muscle fibers associated with the intestine [10,32,33]. Smed-6G10 (6G10) and polyclonal antibody Smed-2G3 (2G3) labeled an extensive network of muscle fibers in the planarian body (shown in the planarian head region in Figure 2A). In the muscle wall, we observed strong 6G10 and 2G3 labeling in circular and diagonal muscle fibers (Figure 2B, arrows and closed arrowheads, respectively), and strong 2G3 labeling in longitudinal fibers (Figure 2B, open arrowheads). Interestingly, 6G10 weakly labeled some longitudinal fibers (bottom insets in Figure 2B), whereas a subset of circular fibers marked by 6G10 were weakly labeled with 2G3 (top insets in Figure 2B). In addition, 6G10 labeled the layer of enteric muscles that surrounds the intestine (Figure 2C, arrow) and the transverse fibers near the intestine (Figure 2C, closed arrowhead). By contrast, 2G3 staining was not detected in this muscle population, but was detected in the dorsoventral fibers of the parenchyma (open arrowhead, Figure 2C). This difference was striking in the mouth region where 6G10 marked the pharyngeal muscles; 2G3 labeled circular fibers surrounding the periphery of the mouth but did not label the pharynx (Figure 2D).

The planarian musculature is composed of myocytes that lack striations but match neither the smooth nor striated muscle types. Planarian muscle fibers differ in their composition of myosin heavy chain proteins (MHC-A and -B) [32,34]. The 6G10 and 2G3 staining patterns were similar to those of MHC-A and -B containing muscle fibers, respectively [32,34]. It has been shown that MHC-A containing muscle fibers are found in the pharynx, enteric muscles, and the circular and diagonal muscle wall fibers in *Dugesia japonica*, similar to what we observed in *S. mediterranea* labeled with 6G10 (Figure 2B-D). By contrast, MHC-B containing muscle fibers are located in body-wall muscles and dorsoventral fibers, but not in enteric muscle fibers [32], which correlates with 2G3 labeling. Similar to MHC-A and -B proteins, our data suggest 6G10 and 2G3 recognize differentially expressed proteins in muscle.

### Smed-1H6 marks axonal projections in the nervous system

**Figure 3:**
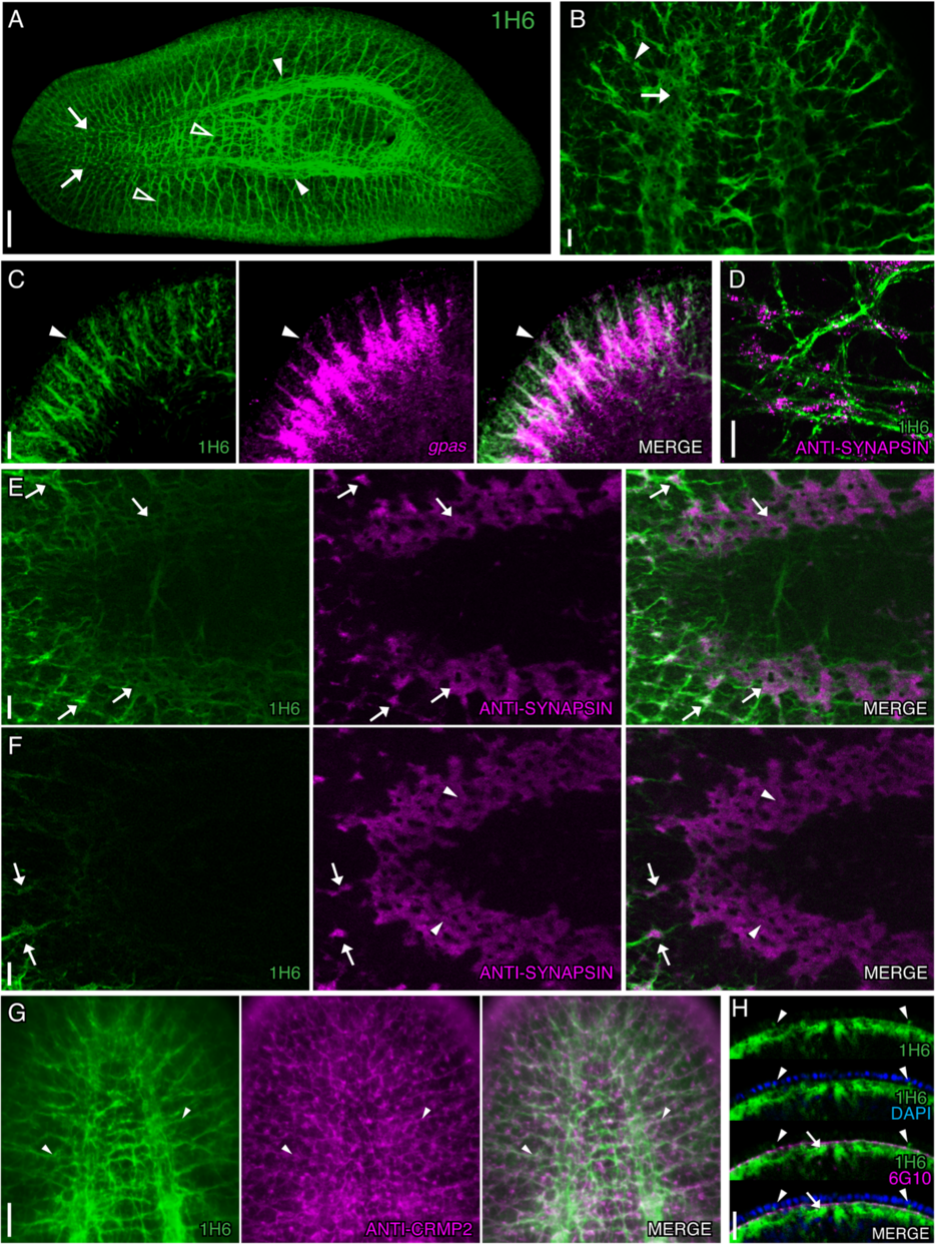
**Smed-1H6 labels CNS and PNS axonal projections. (A-F)** Whole-mount view of intact planarians immunostained with 1H6 (green) in conjunction with other antibodies or FISH to genes indicated in the panels. **(A)** 1H6 labels neural structures in the intact planarian. Arrows mark the anterior end of the ventral nerve cords (VNCs); closed arrowheads mark the VNCs near the pharynx. Open arrowheads highlight transverse and lateral axon branches. **(B)** Higher magnification image of 1H6 staining in the head region shows labeling in the anterior end of the VNCs (arrow) and in lateral branches (arrowhead). **(C)** 1H6 labels *gpas* ^*+*^ (magenta) brain branches in the head. Arrowheads denote one of the co-labeled branches. **(D-F)** Planarians double-labeled with1H6 and anti-SYNAPSIN (magenta). **(D)** High magnification shows that 1H6 labels neuronal projections in close association with SYNAPSIN^+^ synapses. **(E)** Co-labeling in the anterior region of the VNCs. **(F)** 1H6 staining is absent in the neuropil of the cephalic ganglia. Arrows point to examples of 1H6 and anti-SYNAPSIN co-labeling, whereas arrowheads (in **F**) mark the SYNAPSIN^+^ neuropil of the cephalic ganglia. **(G)** 1H6 labels many CRMP2^+^ (magenta) neurons in the intact planarian (shown in the head region, highlighted with arrowheads). **(H)** 1H6, 6G10 (magenta), and DAPI (blue, epidermal nuclei) labeling at the anterior tip of the worm demonstrates that 1H6 labels axon projections within the submuscular plexus. Arrowheads mark 1H6^+^ axons extending between epithelial cells. Images are maximum intensity projections of optical sections, except in **A**. Anterior is to the left in **A**, **E**, and **F** and to the top in **B**-**D** and **G**-**H**. Images were taken to the right side of the pharynx (facing the ventral side of the animal) in **D** and to the left side of the cephalic ganglia in **C**. Scale bars: **(A)** 200 μm; **(B-G)** 20 μm; **(H)** 50 μm.

Smed-1H6 (1H6) labeled the axonal projections in subsets of cells in both the central and peripheral nervous systems. In the CNS, 1H6 labeled the ventral nerve cords (VNCs) (Figure 3A, closed arrowheads), which are known to extend anteriorly through the head region, beneath the cephalic ganglia [35]. 1H6^+^ projections were observed in the anterior tip of the VNCs (Figure 3A and 3B, arrows). 1H6 also labeled the transverse axon branches between the VNCs and the lateral axon branches extending from the VNCs (Figure 3A, open arrowheads). We observed 1H6 labeling in the lateral branches of the cephalic ganglia (Figure 3B, arrowhead); these axon projections are known to extend to the sides of the head where they penetrate the epidermis in sensory neuron-rich areas [36,37]. To confirm 1H6 labeling in these branches, we processed planarians for 1H6 immunolabeling and *in situ* hybridization to *G protein α-subunit* (*gpas*), which marks distal lateral branch neurons [7]. We found that 1H6 strongly co-labeled with *gpas* (arrowheads in Figure 3C)*,* suggesting that 1H6 binds an antigen found in the axonal projections of sensory neurons.

Our initial observations were that 1H6 did not label the neuropil region of the cephalic ganglia; thus, we co-stained 1H6-labeled planarians with the pan-neural antibody anti-SYNAPSIN [6]. We found that while 1H6 labeling corresponded with SYNAPSIN expression along many neural projections throughout the nervous system (Figure 3D; arrows in Figure 3E) 1H6 labeling was absent in the SYNAPSIN-dense neuropil of the cephalic ganglia (Figure 3F, arrowheads).

Axons labeled with 1H6 partially overlapped with neural projections that were positive for anti-Collapsin Response Mediator Protein 2 (anti-CRMP2) throughout the body (examples in the head region are highlighted with arrowheads in Figure 3G). CRMP2 is a cytosolic phosphoprotein found across the metazoans with high expression during neural development and retained expression in mature neurons, and is implicated in neurite outgrowth, synaptic assembly, calcium channel regulation, and neurotransmitter release [38]. We found that a commercially available antibody designed against human CRMP2 marked neurons throughout the planarian CNS and peripheral nervous system (PNS, both cell bodies and projections) and recently reported that CRMP2^+^ neurons co-label with the neural markers β-TUBULIN and *choline acetyltransferase* (*ChAT*) [39]. Additionally, we observed that some 1H6^+^ cells were positive for *prohormone convertase 2* (*pc2*; arrowheads in Additional file 2: Figure S1); *pc2*, which encodes a protease that is essential for processing neuropeptide precursor proteins to their mature forms, is found in a subset of cells throughout the planarian nervous system [5,40].

Planarians are enveloped with a monolayer of ciliated epithelium, beneath which lies a thin (0.1-0.2 μm) nerve net, referred to as the subepidermal nerve plexus [10,41]. The fibers in this plexus extend through the basement membrane of the epidermal layer, forming intra-epithelial fibers [41]. Proximal to the subepidermal plexus lies the muscle wall, followed by a submuscular nerve net (submuscular nerve plexus) [41]. These plexuses are part of the peripheral nervous system in planarians [41]. To determine if these plexuses contained 1H6^+^ projections, we co-stained 1H6-labeled planarians with 6G10 and DAPI to label the contractile muscle layer and visualize the epithelial nuclei, respectively. 1H6^+^ fibers were seen in the submuscular plexus (Figure 3H, arrows) and in projections extending between epithelial cells (arrowheads in Figure 3H). To resolve labeling in the subepidermal plexus, we observed 1H6-labeled planarians from the ventral surface through the submuscular plexus at 0.44 μm intervals. We noted projections extending between and past the epithelial nuclei (Additional file 3: Figure S2, 0.44 μm to 4.40 μm) and sparse mesh-like fibers as the epithelial nuclei disappeared from the field-of-view (4.84 μm and 5.28 μm in Additional file 3: Figure S2) indicating labeling of the subepidermal plexus. 1H6^+^ fibers projected through the muscle layers, etching a pattern in the negative impressions of the muscle fibers: circular, longitudinal, diagonal, and finally the last layer of longitudinal fibers of the muscle wall (seen from 5.28 μm to 9.68 μm in Additional file 3: Figure S2). The thicker, more clearly defined meshwork of the submuscular plexus was detected below the final muscle layer (shown from 10.12 μm through 11.88 μm in Additional file 3: Figure S2). It will be interesting to identify the epitope recognized by 1H6 to resolve if it labels a subpopulation of neurons in the central and peripheral nervous systems or a specific cellular compartment found in most neurons.

### Smed-2C4 is expressed in discrete cells in the regeneration blastema and a subset of neurons and intestinal cells

**Figure 4:**
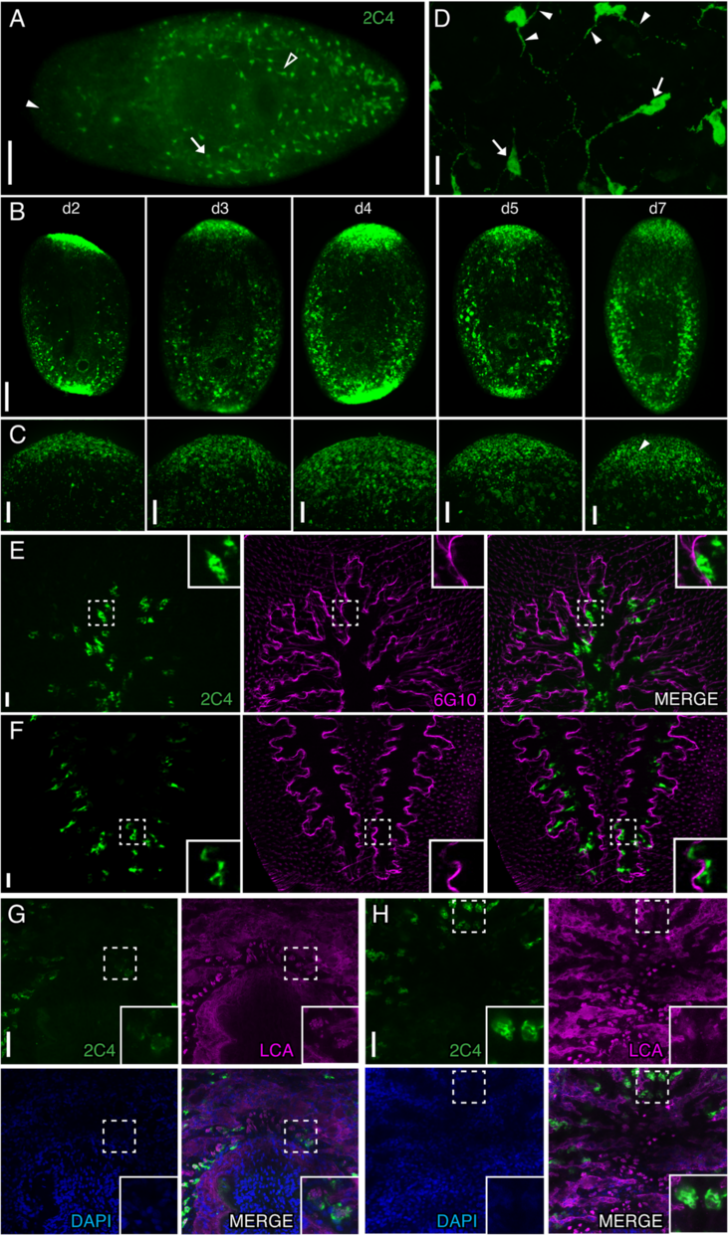
**Smed-2C4 labels multiple cells with distinct morphologies and anatomical locations. (A-F)** Whole-mount staining of intact planarians or regenerating planarians with 2C4 (green) and with either 6G10 (magenta) in panels **E** and **F** or *Lens culinaris* agglutinin lectin (LCA, magenta) in panels **G** and **H**.** (A)** 2C4 labels multiple distinct cell types in the intact worm. Closed arrowhead indicates an example of a 2C4-S cell. Open arrowhead highlights a 2C4-N cell. Arrow indicates a large round 2C4-I cell. **(B, C)** 2C4 labels the anterior and posterior blastema during regeneration in 2, 3, 4, 5, and 7 dpa trunk regenerates. Higher magnification images of the anterior blastemas are shown in **C**. Arrowhead highlights an example of the 2C4-S cells seen throughout regeneration. **(D)** Magnified image of 2C4-N cells. Arrows denote the large cell bodies and arrowheads indicate their projections. **(E, F)** 2C4-I cells are located within the anterior (shown in **E**) and posterior (shown in **F**) intestinal branches (delineated by labeling of the intestinal wall musculature with 6G10). **(G, H)** 2C4 is expressed in a subset of goblet cells marked with LCA. Strongly labeled LCA^+^ cells immediately anterior to the pharynx were weakly labeled with 2C4 (shown in **G**). In contrast, strongly labeled 2C4 cells in anterior secondary branches were weakly labeled with LCA (shown in **H**). Dashed boxes in **E**-**G** indicate the areas shown in the inset images. Images are maximum intensity projections of optical sections except for **A** and **B**. Anterior is to the left in **A** and to the top in **B**-**G**. Image **D** was acquired adjacent to the pharynx. Scale bars: **(A, B)** 200 μm; **(C, E-H)** 50 μm; **(D)** 20 μm.

We observed multiple distinct cell morphologies (which we refer to as cell types hereafter) throughout the planarian body that labeled with Smed-2C4 (2C4) (Figure 4A). The first cell type (2C4-S cells) had cytoplasmic 2C4 labeling and small cell bodies approximately 4.4 μm in diameter (N = 123 cells measured from 10 worms). We observed that 2C4-S cells were weakly labeled with 2C4 near the epidermis throughout the intact worm (Figure 4A, closed arrowhead; Additional file 4: Figure S3A, arrows) but strongly labeled within the regeneration blastema (Figure 4B and C). To explore whether these cells were secretory, we performed co-labeling experiments with the lectin wheat germ agglutinin (WGA), which labels a subset of secretory cells [20] and found that 2C4-S cells were not WGA^+^ (Additional file 4: Figure S3A). In order to determine when the protein recognized by 2C4 is expressed during regeneration, we amputated animals anterior and posterior to the pharynx to analyze the presence of 2C4-S cells during 7 days post-amputation. 2C4^+^ cells were detected in both the anterior and posterior blastemas at all the timepoints we examined (Figure 4B-C).

The second cell type labeled by 2C4 consisted of ventrally located oblong cells (2C4-N cells) of approximately 10.7 μm in diameter (N = 58 cells measured from 9 worms through the longest path of the cell body) (Figure 4A, open arrowhead; Figure 4D, arrows). These cells have bipolar projections and resemble the large multipolar neurons observed in flatworms using silver nitrate staining and Lucifer Yellow dye [42,43]. The projections from these cells extend both laterally and longitudinally (arrowheads in Figure 4D). We found that 2C4-N cells were located on the ventral side of the planarian, excluding the head region (Figure 4A, open arrowhead). Similar to 2C4-S cells, these cells did not co-label with WGA (Additional file 4: Figure S3B.) Based on morphology and their large size, we hypothesize that 2C4-N cells are likely neurosecretory cells. Future experiments should test if 2C4 co-labels cells expressing neuropeptide genes in planarians [40].

The third cell type observed with 2C4 labeling was a large 18.4 μm in diameter (N = 83 cells measured from 10 animals) round cell with large cytoplasm that appeared to be located in or near the intestine (2C4-I cell, arrows in Figure 4A; insets in Figures 4E-F). To determine the relative location of these cells, we co-labeled 2C4-stained planarians with 6G10 to visualize the enteric muscles, which serve as a boundary between the intestinal epithelium and the mesenchyme (as described in [11]). We found that 2C4-I cells were located within the anterior and posterior branches of the intestine (Figure 4E- F). We further explored the identity of these cells by performing co-labeling experiments with the lectin *Lens culinaris* agglutinin (LCA), which labels goblet cells [20]. We found that a subset of 2C4-I cells were indeed LCA^+^ goblet cells (Figure 4G). Strikingly, we found that 2C4-I^+^ cells in the secondary and tertiary intestinal branches had fainter LCA expression (Figure 4H, inset), whereas the goblet cells with strongest LCA expression anterior to the pharynx were weakly positive for 2C4 (Figure 4G, inset). Because 2C4 labels large neuronal cells surrounding the pharynx and cells within the intestine, the protein that 2C4 recognizes may be highly expressed in cells involved in secretion; however, this question will only be definitively answered upon identification of the 2C4 epitope.

We found that 2C4 labeling was highly dependent on the fixation protocol used (Figure 1C). Whereas 2C4-I and -N cells could be seen with a multitude of fixation protocols, labeling of 2C4-S cells within the blastema was absent or very difficult to detect in fixations using HCl for the initial kill step or with inclusion of Proteinase K treatment.

### Smed-5B1 marks ciliated cells

**Figure 5:**
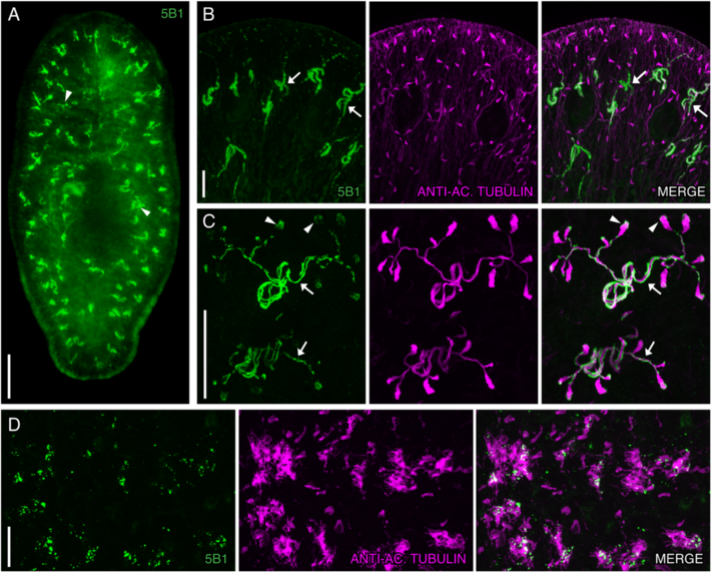
**Smed-5B1 labels ciliated cells. (A-C)** Whole-mount view of intact planarians immunostained with 5B1 (green), and co-labeled with anti-Acetylated Tubulin (magenta) and DAPI (blue) in panel **B** and anti-Acetylated Tubulin (magenta) in **C**.** (A)** 5B1 labels protonephridial tubules. Arrowheads indicate examples of protonephridia. **(B)** Image of the head region showing that 5B1 labels the protonephridial tubules that are positive for Acetylated Tubulin. Arrows show examples of protonephridia tubules. **(C)** A higher magnification image of protonephridia. Arrows point to examples of 5B1-labeled tubules. Arrowheads show examples of 5B1 labeling in flame cells. **(D)** 5B1is shown to label in immediate proximity to anti-Acetylated Tubulin in the dorsal ciliated stripe. Images are maximum intensity projections of optical sections except for in **A**. The anterior of the animal is to the top in **A** and **B** and to the left in **D**. Scale bars: **(A)** 200 μm; **(B, C)** 50 μm; **(D)** 20 μm.

Staining planarians with Smed-5B1 (5B1) revealed a pattern strikingly similar to those observed with anti-Acetylated Tubulin and *in situ* hybridizations against protonephridial markers (arrowheads in Figure 5A) [14,15]. The planarian protonephridial system, analogous to the metanephridial systems found in vertebrates [44], maintains osmoregularity and excretes waste. Protonephridia in planaria consist of fenestrated, ciliated flame cells, which connect to tubules that are ciliated proximal to the flame cells and are non-ciliated distal to the flame cells [14,15]. Protonephridia are located along the majority of the length of the planarian body. Anti-Acetylated Tubulin labels the flame cells and ciliated tubules of protonephridia as well as all other ciliated structures in planarians [25]. Therefore, we tested if 5B1 would co-label with anti-Acetylated Tubulin. We found that 5B1 labeled the tubules of the protonephridia, exterior to the cilia, in a pattern consistent with labeling of the ciliated tubule cells’ cytoplasm or membrane (arrows in Figure 5B and C). Labeling was also observed surrounding Acetylated Tubulin labeling at the flame bulb, which is also highly ciliated (arrowheads in Figure 5C). This observation led us to explore whether 5B1 was associated with other ciliated cell types. Planarians have a high abundance of ciliated cells in the epithelium on their ventral surface and in a discrete stripe running along the dorsal anteroposterior axis; these structures are positive for Acetylated Tubulin [24,45,46]. We detected 5B1 labeling within the dorsal ciliated stripe and the ventral ciliated epithelial cell surface (dorsal staining shown as a representative example in Figure 5D).

### Smed-1D9 labels the nucleus of a subset of progenitors and cells in close proximity to the central nervous system

**Figure 6:**
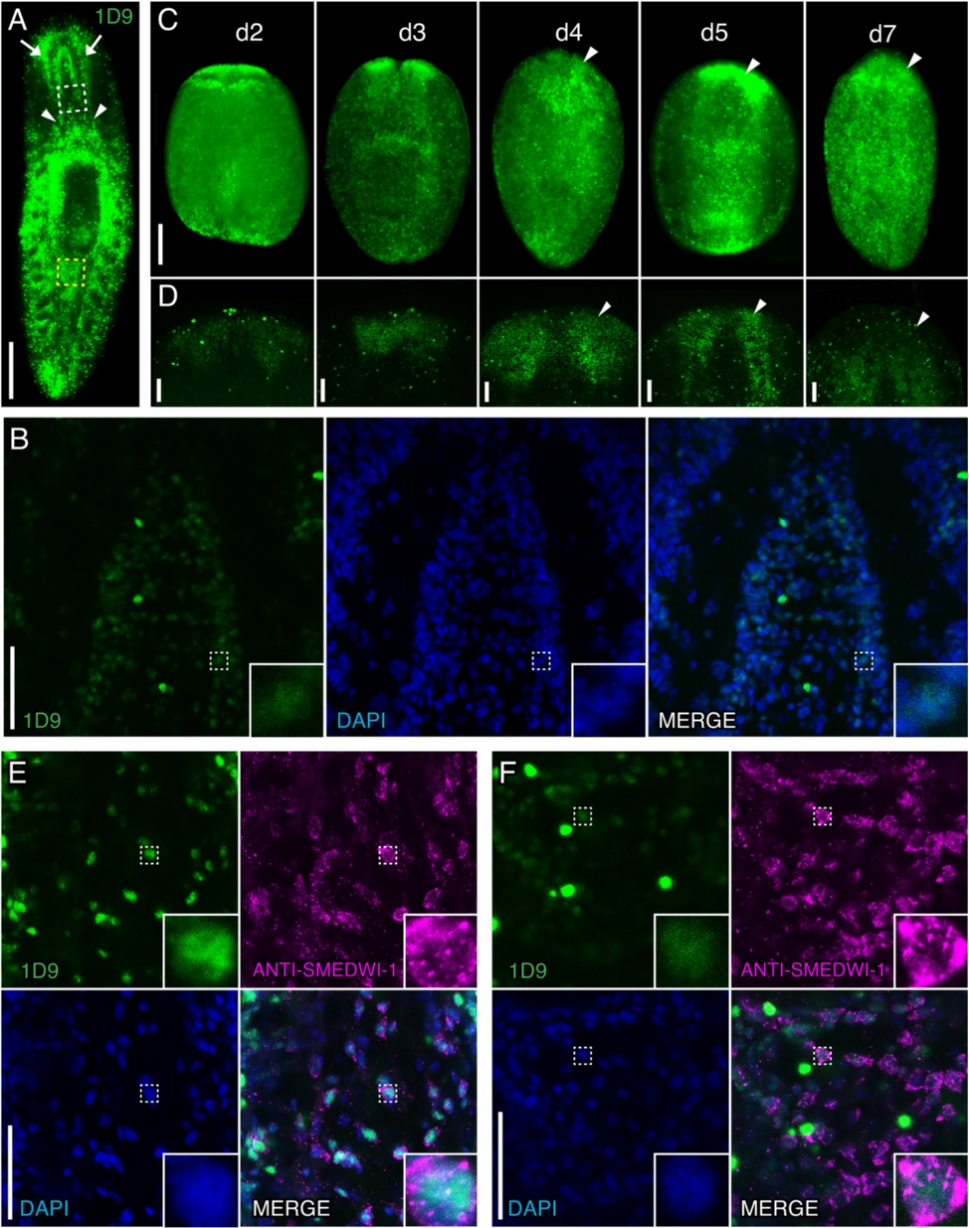
**Smed-1D9 labels the CNS and neoblast progeny. (A-D)** Whole-mount view of intact planarians or regenerating planarians immunostained with 1D9 (green), and counterstained with DAPI (blue) in **B** and stained with anti-SMEDWI (magenta) in **E**-**F**. **(A)** 1D9 staining in the intact planarian reveals labeling surrounding the cephalic ganglia (arrows) and ventral nerve cords (arrowheads), and in the mesenchyme. Dashed boxes indicate the regions shown in **E** (white), and **F** (yellow). **(B)** Higher magnification image of the cephalic ganglia demonstrates that 1D9 labels the nucleus (counterstained with DAPI). **(C, D)** 1D9 labels the anterior and posterior blastema during regeneration in 2, 3, 4, 5, and 7 dpa trunk regenerates. The morphology of the nascent cephalic ganglia is apparent by 4 dpa (indicated by arrowheads). **(D)** View of the anterior regeneration blastema. **(E)** Image of the area proximal to the posterior end of the cephalic ganglia in an intact planarian shows a large population of 1D9^+^ cells co-labeled with anti-SMEDWI. **(F)** Image of the area immediately posterior to the pharynx showing the presence of 1D9^+^ cells co-labeled with anti-SMEDWI. Dashed boxes indicate the area shown in the insets. Images are maximum intensity projections of optical sections except for **A** and **C**. Anterior is to the top in all images. Scale bars: **(A)** 200 μm; **(B, D-F)** 50 μm; **(C)** 100 μm.

Smed-1D9 (1D9) marked cells throughout the mesenchyme and surrounding the cephalic ganglia (Figure 6A, arrows) and VNCs (Figure 6A, arrowheads). Counterstaining with DAPI revealed that 1D9 labeled cell nuclei (Figure 6B, inset). The labeling pattern in the mesenchyme was reminiscent of staining for neoblasts [47] and their early progeny [48]. During our initial screenings, we noted that 1D9 strongly labeled the regeneration blastema; thus, we examined the pattern of 1D9 expression during regeneration. We found that 1D9 labeled the blastema at 2 dpa through one week of regeneration in a pattern reminiscent of the nascent cephalic ganglia (arrowheads in Figure 6C and 6D), although we do not think the staining is exclusively in neurons. Co-labeling with additional markers will be necessary to confirm which cell types label with 1D9 during regeneration.

The neoblast and progenitor-like labeling pattern and the presence of 1D9^+^ cells in the regeneration blastema led us to explore the possibility that 1D9 labels either neoblasts or progenitors. To test this, we performed co-labeling experiments with 1D9 and anti-SMEDWI, which labels both neoblasts and their progeny [23]. We first examined the region near the cephalic ganglia in the intact worm and detected many co-labeled cells (inset in Figure 6E). Next, we inspected the animals for co-labeling in the mesenchyme, immediately posterior to the pharynx, which is rich in neoblasts. Intriguingly, we observed that fewer SMEDWI^+^ cells co-labeled with 1D9 in this region and also noticed that the levels of 1D9 expression were lower when compared with double-labeled cells proximal to the cephalic ganglia (Figure 6F, N = 6 animals). Further experimentation and quantitative analysis will be required to resolve the differential expression of 1D9 throughout the animal. The expression of genes known to play roles in neural differentiation has been observed in both differentiated cells and their progenitors [49-51]. Therefore, we hypothesize that 1D9 recognizes a protein present in progenitors and cells associated with the nervous system.

### Smed-6C8 is expressed in cells within the planarian intestine and the regeneration blastema

**Figure 7:**
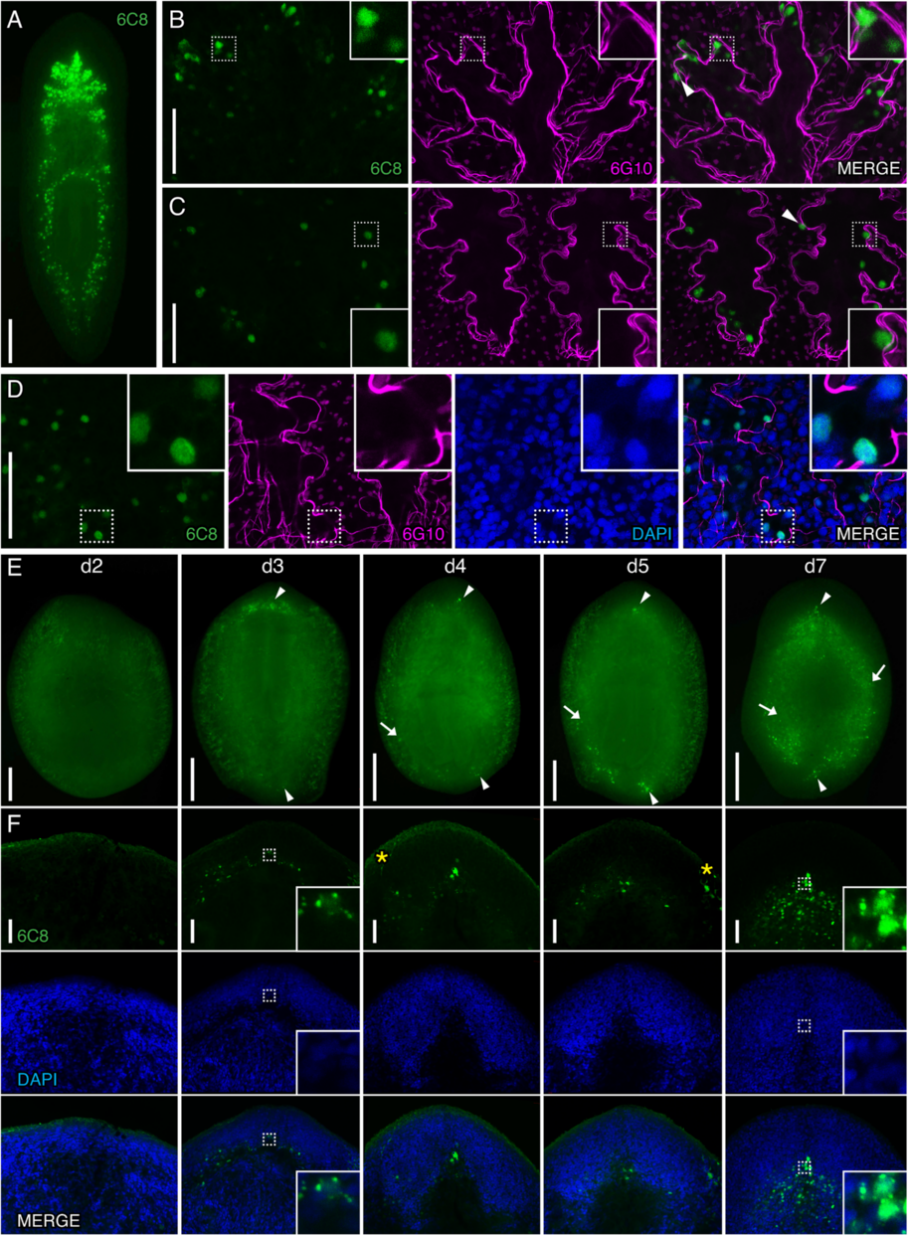
**Smed-6C8 labels intestinal cells and cells near the regeneration blastema. (A-C)** Whole-mount view of intact or regenerating planarians immunostained with 6C8 (green) and co-labeled with 6G10 (magenta) in panels **B**-**D** and/or counterstained with DAPI (blue) in **D** and **F**.** (A)** 6C8-labeled cells near or within the intestine. **(B, C)** 6C8 cells are located within the anterior (shown in **B**) and posterior (shown in **C**) intestinal branches or in contact with the enteric musculature wall, which is delineated with 6G10 labeling. Examples of cells observed outside the enteric muscle boundary are highlighted with arrowheads. **(D)** 6C8 labels cell nuclei (observed within the boundary of the enteric musculature). **(E, F)** 6C8^+^ cells appear near the anterior and posterior regeneration blastemas at 3 dpa and are detected in the blastema at all later timepoints assayed (examples highlighted with arrowheads). By 5 dpa, examples of 6C8^+^ cells were detected far from the blastemas (arrows). Higher magnification image of the anterior blastema shown in **F**. Yellow asterisks indicate non-specific labeling of secretory cells. Dashed boxes indicate the area of the high magnification image shown in the insets. Images are maximum intensity projections except for **A** and **E**. Anterior of is to the top in all images. Scale bars: **(A, E)** 200 μm; **(B-D, F)** 50 μm.

Smed-6C8 (6C8) labeled cells in a punctate pattern throughout the planarian body that resembled the shape of the intestinal branches (Figure 7A). Therefore, we performed co-labeling experiments with 6G10 to visualize the enteric musculature and determine if 6C8 marks intestinal cells. These experiments showed that 6C8^+^ cells were generally located on the luminal side of the enteric muscle wall in both the anterior and posterior of the animal (Figures 7B and C). However, some 6C8^+^ cells were detected outside, but still associated with the enteric muscular boundary (arrowheads in Figures 7B and C; Additional file 5: Figure S4). We counterstained samples with DAPI and found that 6C8 labeling was located in the nucleus of cells (Figure 7D, inset). Because 2C4 also labeled an intestinal cell population, we examined if the 2C4 and 6C8 epitopes were expressed in the same cells or in different cell populations. When we performed co-labeling experiments, we noted that 2C4 and 6C8 marked distinct intestinal cell populations (Additional file 6: Figure S5A). Furthermore, 6C8 was not expressed in LCA^+^ goblet cells (Additional file 6: Figure S5B).

Intestinal cells are derived from neoblasts that divide outside of the intestine [11]. Our experiments showed 6C8 labels a cell population adjacent and within the intestinal musculature boundary. Thus, we hypothesized that 6C8 labels cells that are specified to become intestinal cells in the mesenchyme and are in the process of incorporating into the intestinal epithelium. To evaluate this possibility further, we examined 6C8 expression during regeneration. We amputated planarians anterior and posterior to the pharynx and observed 6C8 labeling between 2 and 7 days of regeneration (Figure 7E and 7F). 6C8-labeled cells were difficult to detect or completely absent in 2 dpa regenerates in the blastema and throughout the worm (Figure 7E). In contrast, we readily detected strong expression of 6C8 near the anterior and posterior regeneration blastemas from 3 to 7 dpa (Figure 7E, arrowheads; Figure 7F, insets); we also noted cells with 6C8 expression in a cytoplasmic punctate pattern (insets in Figure 7F). Interestingly, beginning at 4 dpa we observed 6C8 labeling outside of the blastema in a pattern resembling the intestinal branches (arrows in Figure 7E). Extensive remodeling of the intestine is required to restore proper intestinal morphology following amputation [11]. The presence of 6C8^+^ cells within the intestinal musculature boundary and their location during regeneration suggests to us that these cells represent differentiating intestinal cells. This possibility should be tested further with BrdU pulse/chase experiments to determine if 6C8^+^ cells co-label with BrdU within the enteric muscle boundary [11].

### Smed-5E12 is strongly expressed in cells in the regeneration blastema

**Figure 8:**
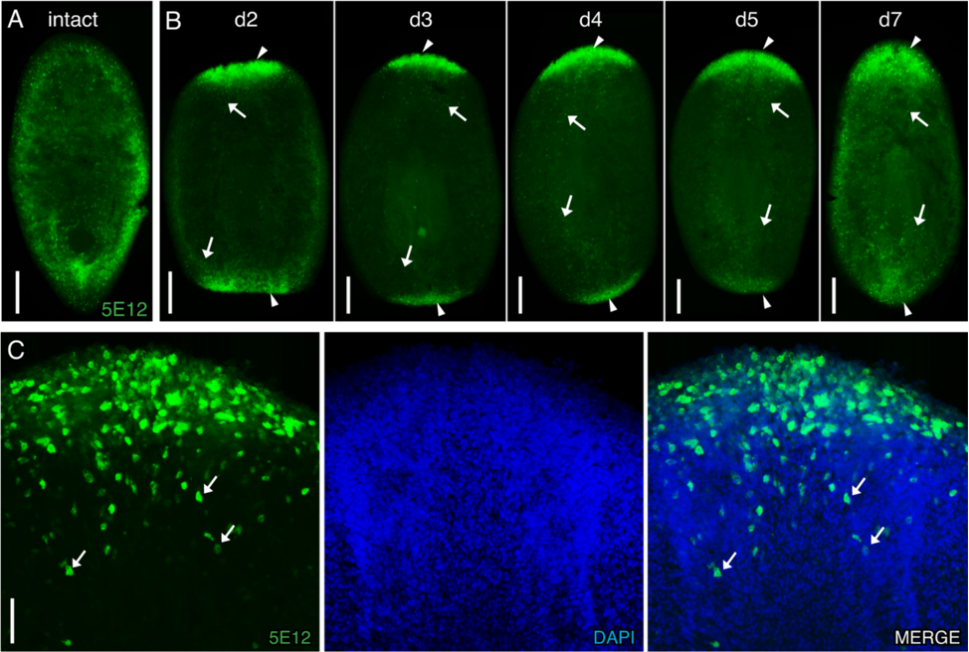
**Smed-5E12 labels the regeneration blastema.** Whole-mount images of intact or regenerating planarians immunostained with 5E12 (green) and counterstained with DAPI (blue) in panel **B**.** (A)** 5E12-labeled cells detected throughout the body of an intact animal. **(B)** 5E12 labels the anterior and posterior blastemas in regenerating trunk fragments stained over the course of 7 days post-amputation (marked by arrowheads), and cells proximal to the regeneration blastemas or throughout the body (marked by arrows at 2–7 dpa). **(C)** Higher magnification image of 5E12^+^ cells within a 3 dpa regeneration blastema and posterior to the blastema (arrows). Images in **C** are maximum intensity projections of optical sections. Anterior is up in all images. Scale bars: **(A, B)** 200 μm; **(C)** 50 μm.

Smed-5E12 (5E12) was not readily detected in intact planarians. We observed a punctate expression pattern in some animals, but this was inconsistent (i.e., the pattern was completely absent from some of the samples). Therefore, it was difficult to discern 5E12 labeling in defined cell types or tissues (Figure 8A). By contrast, when we amputated planarians, fixed, and immunolabeled them at 3 dpa, we observed strong 5E12 labeling in anterior and posterior regeneration blastemas (Figure 8B). To determine the expression of this antibody during the course of regeneration we fixed and stained regenerating trunk fragments at 2–7 dpa and observed 5E12 labeling in the blastema at all the time points tested (Figure 8B, arrowheads). It was interesting to note that expression was seen in a population of cells proximal to the blastema in regenerating animals (highlighted posterior to the 3 dpa anterior regeneration blastema in Figure 8B and C, arrows), suggesting that 5E12 labels a progenitor population. Thus, 5E12 can serve as a marker of the blastema following injury or amputation. It will be interesting to determine if 5E12 can mark the formation of ectopic structures such as generation of supernumerary heads that follow RNAi knockdown of *β*-*catenin-1* in intact animals [52-54]. In addition, the fixation protocol for this antibody may require further optimization to stain or preserve the epitope in non-injured animals, and additional co-labeling experiments will be needed to investigate the identity of the 5E12^+^ blastema cells.

### Visualization of the *slit* RNAi midline collapse phenotype with the newly generated mAbs

**Figure 9:**
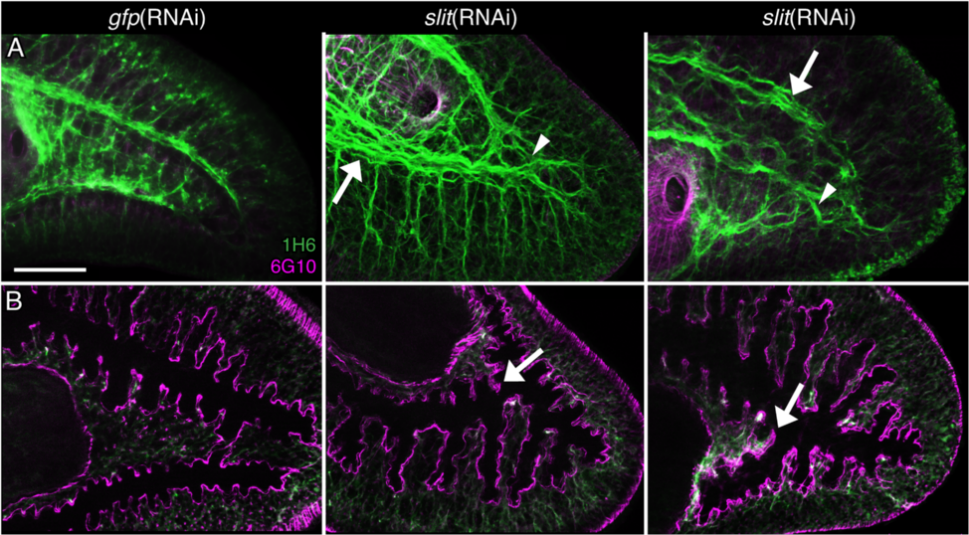
**Smed-1H6 and -6G10 co-labeling reveal the complexity of the midline collapse phenotype following slit RNAi. (A-B)** Whole-mount view of a planarian bisected sagittally and immunostained 15 dpa with 1H6 (green) and 6G10 (magenta). **(A)** Image of the ventral nerve cords (VNCs) shows *slit(RNAi)* worms with intersecting VNCs (arrowheads, 3/5 *slit(RNAi)*, 0/5 *gfp(RNAi)* animals), as well as *slit(RNAi)* worms with widely spaced VNC tracks (arrows, (5/5 *slit(RNAi)*, 0/5 *gfp(RNAi)* animals). **(B)** Image of the enteric muscles delineating the intestine illustrates fusion of the intestinal branches posterior to the pharynx in *slit(RNAi)* animals (arrows, 5/5 *slit(RNAi)* animals, 1/5 *gfp(RNAi)* animals). Images are maximum intensity projections of optical sections. Anterior is to the left in all images. Scale bar: 200 μm.

As a proof of concept, we revisited the knockdown of *Smed-slit* [55] to test the utility of our new mAbs in characterizing regeneration defects present following RNAi. *Smed-slit* (*slit*) encodes a conserved axon-guidance glycoprotein that has a repellent role at the midline and is necessary for patterning of the major organ systems in planarians with respect to distance from the midline [55]. Loss of *slit* by RNAi causes planarians to regenerate mispatterned VNCs and fused primary intestinal branches in the posterior of the animal [55]. We knocked down *slit,* amputated the animals along the sagittal plane, and labeled regenerates with 6G10 and 1H6 to visualize the intestinal muscle boundary and the VNCs, respectively. We observed widely spaced axonal tracks in the VNCs posterior to the pharynx in all *slit(RNAi)* animals (Figure 9A, arrows) giving the appearance of either the formation of multiple VNCs or a diversion of a subset of VNC axonal tracks from the midline. The VNCs were collapsed at the midline in the posterior of all *slit(RNAi)* animals (Figure 9A, arrowheads), whereas the nerve cords regenerated normally in the control animals. We also observed that the primary intestinal branches in the posterior regions of *slit(RNAi)* animals appeared to be connected (Figure 9B, arrows), unlike those in *gfp(RNAi)* animals, which regenerated two posterior primary gut branches. Thus, use of these new mAbs allowed robust visualization of morphological defects present after RNAi.

## Conclusions

We have produced new monoclonal antibodies that can be used to study tissue regeneration, structure, and function in planarians. With the exception of polyclonal Smed-2G3, these antibodies were deposited to the Developmental Studies Hybridoma Bank. We have demonstrated that these antibodies label diverse tissues in planarians and can be useful for examining phenotypes following RNAi. In addition, we determined that the labeling efficiency of antibodies used for whole-mount staining of planarians greatly depends on the fixation protocols. Many of the mAbs that we generated did not display any discernable staining pattern when treated with subsets of the reagents used in the eight formaldehyde fixation protocols we tested (Figure 1C). The observation that most of the antibodies do not work well with Carnoy’s fixative is likely due to the fact that these antibodies were generated against cells fixed with formaldehyde. Further optimization of these protocols could prove helpful for future experiments that warrant co-labeling with currently incompatible antibodies.

Future studies will examine the epitopes detected by these mAbs, which could potentially increase their usefulness as markers for phenotypic analysis or other applications. It will be interesting to determine if these antibodies label other species of planarians and the hermaphroditic strain of *S. mediterranea.* As an initial test, we performed labeling experiments with three of these antibodies (1H6, 2C4, 6G10) in hermaphrodites. As expected, we found that these mAbs produced the same staining patterns observed in the asexual strain, but with limited penetrance throughout the body and increased background signal (data not shown). Additional modifications to the fixation protocols will be necessary to use the mAbs with larger animals as in previous studies [21,40,56]. In conclusion, these antibodies should help to expand the molecular toolkit available for studies using planarians.

## Methods

All experiments involving mice were performed at QED Bioscience Inc. (San Diego, CA) in strict accordance with the policies and guidelines of the Public Health Service Policy on Humane Care and Use of Laboratory Animals and the Institutional Animal Care and Use Committee (IACUC) of QED Bioscience Inc. (San Diego, CA). Immunizations were performed in accordance with QED Bioscience’s IACUC-approved protocol (SOP #22).

### Planarian culture

Asexual *Schmidtea mediterranea* (CIW4) were maintained in ultrapure water containing either Instant Ocean (Spectrum brands) at 0.21 g/l, 0.83 mM MgSO_4_, 0.9 mM CaCl_2_, 0.04 mM KHCO_3_, and 0.9 mM NaHCO_3_, or Montjuïc salts as previously described [22]. Unless otherwise noted, planarians 3–5 mm in length that were starved for at least 5 days were used for all experiments.

### Dissociation of cells for immunization

Three days prior to the initial amputation, animals were fed to increase cell proliferation [57]. Animals were amputated anterior to the pharynx, and fragments containing the tail were allowed to regenerate for approximately 3 days and then washed in Calcium Magnesium Free media (CMF) [58], followed by a second wash in CMF with 30 μg/ml trypsin inhibitor (CMF+TI [T9253, Sigma]) prior to amputation immediately posterior to the blastema. The blastemas were collected and incubated in CMF+TI at 4°C for 10 minutes. After removing the CMF+TI, blastemas were diced and transferred into a tube with CMF plus 2 U/ml trypsin (T0303, Sigma) and incubated, while rocking, at 4°C for 1–3 hours (triturated every 30–45 minutes with a transfer pipette). Once the tissues neared complete dissociation, the samples were triturated with a 1000 ml micropipette tip. The cell suspension was spun down at 300 × g at 4°C for 5 minutes, and the pellet was re-suspended in CMF and passed through a 50 μm mesh filter. The cell suspension was centrifuged as above and re-suspended in 4% formaldehyde (FA) for 10 minutes at room temperature, after which the cells were centrifuged at 300 × g for 5 minutes and re-suspended in Phosphate Buffered Saline (PBS). Cell counts were performed on a Petroff-Hausser counter to determine cell density.

### Mouse immunizations

Five Balb/c mice were injected a total of 5 times over the course of 7 weeks. The initial immunization and first boost contained a mixture of 5 × 10^5^ fixed cells suspended in 250 μl mixed with 250 μl of a propriety adjuvant (QED Bioscience). All subsequent boost injections contained 5 × 10^5^ cells in PBS. Following all injections, one mouse was selected and its spleen was harvested. Blood sera were collected and tested for cross-reactivity to planarian epitopes after each booster administration using FA-fixed planarians. For these tests, planarians were killed in 2% HCl in PBS for 3 minutes followed by a 4 hour incubation at 4°C in 4% FA in PBSTx (PBS + 0.3% Triton X-100) and bleached overnight in a 6% H_2_O_2_ solution in PBS. Prior to overnight incubation in blood sera, the worms were blocked for 4 hours in PBSTB (PBSTx + 1% Bovine Serum Albumin [Jackson ImmunoResearch Laboratories]). After extensive washing with PBSTx, worms were blocked again for 1 hour in PBSTB and incubated in Alexa Fluor 488 goat-anti-mouse IgG (1:400 in PBSTB, [A11029, Life Technologies]) overnight at 4°C and then washed again with PBSTx the next day.

### Hybridoma screen and antibody production

At QED Bioscience Inc. (San Diego, CA), spleenocytes from the immunized mice were fused with myeloma cells, Species F0 or P3X63Ag8U.1. The resulting hybridomas were expanded and screened to generate a library of polyclonal parental hybridomas. From these parental lines, further expansion and screening allowed generation of monoclonal hybridoma lines producing antibody. Cells were cultured in high glucose-DMEM with sodium pyruvate, supplemented with 2% L-glutamine, 10% FBS and 1% penicillin-streptomycin. For selected monoclonal lines, additional purification of the antibody was carried out. *In vitro* production was performed for the following antibodies: 1D9, 1H6, 2C4, 5E12, and 6G10. The 1D9 and 2C4 antibodies were affinity purified. After the initial testing, the monoclonal cell lines were submitted to the DSHB.

### Immunostaining

For all images shown, we performed labeling experiments with at least three worms and visualized similar staining patterns using an optimal fixation protocol for each antibody (see Additional file 1 and summary in Figure 1C) unless variations were made for co-labeling experiments (described below). Fixations for whole-mount immunostaining were optimized using eight modifications to a formaldehyde fixation protocol developed for *in situ* hybridizations [19]. Briefly, animals were killed in either cold 2% HCl or 5% N-acetyl cysteine in PBS for 5 minutes. Animals were then fixed for either 15 minutes at room temperature or 6 hours at 4°C in 4% FA in PBSTx. To enhance possible presentation of epitopes, some protocols added a reducing step (50 mM DTT, 1% NP-40, 0.5% SDS for 5 to 10 minutes at 37°C). Animals were bleached overnight in 6% H_2_O_2_ diluted either in PBSTx (aqueous) or methanol (anhydrous after stepping through 50% methanol) under direct light. The next morning, if bleaching was performed in methanol, animals were re-introduced into PBSTx through a 50% methanol intermediate and washed with PBSTx. Some protocols included a Proteinase K treatment (2 μg/ml Proteinase K, 0.1% SDS in PBSTx for 10 minutes at room temperature) followed by a post-fix step in 4% FA for 10 minutes at room temperature. Animals were washed twice with PBSTx before blocking for 4 hours at room temperature in PBSTB. Hybridoma supernatant was either added directly to the samples or diluted in PBSTB and incubated overnight at 4°C. The following day, animals were washed extensively with PBSTx, then blocked in PBSTB for 1 hour before an overnight incubation at 4°C in either goat-anti-mouse IgG+IgM-horse radish peroxidase (HRP) (1:1000 [Life Technologies]) or goat-anti-mouse IgG-HRP (1:1000 [Life Technologies]) in PBSTB. The following day, animals were washed extensively and then incubated in FITC-tyramide (1:1000 in PBSTI [PBSTx + 10 mM imidazole]) for 30 minutes and developed in FITC-tyramide in PBSTI containing 0.015% H_2_O_2_ for 5 minutes. Alternatively, animals were developed as previously described [18], with the following modifications: animals were incubated for 5 minutes at room temperature in 0.1 M borate buffer, pH 8.5, with 0.1% Tween-20 and then for 10 minutes at room temperature in 0.1 M borate buffer, pH 8.5, with 0.1% Tween-20, 0.003% H_2_O_2_, 2% dextran sulfate, 1:250 FITC-tyramide. An additional 0.003% H_2_O_2_ was added and incubated for 10 minutes at room temperature. Animals were then washed extensively with PBSTx. Where indicated in the Results section, worms were counterstained with DAPI (0.05 μg/ml) by including it with the secondary antibody incubation step or subsequent overnight incubation at 4°C.

### Double-immunostaining experiments

Immunostaining was carried out as described above, except for the following modifications. To double label planarians with 1H6 and 6G10, the antibodies were detected using either Zenon Alexa Fluor 488, 568, or Zenon HRP IgG1 Labeling Kits (Z-25002, Z-25006, Z-25054, Life Technologies). The ratios of the antibody, kit component A, and kit component B used were 2:1:1; samples were incubated according to manufacturer’s instructions. Labeled antibodies were then diluted to 1:250 in PBSTB. Double-labeling with 2C4 and 6C8 was performed as described above for 2C4; then TSA signaling was quenched with 10 μg/ml Proteinase K in PBSTx with 0.1% SDS for 10 minutes at room temperature. Animals were post-fixed in 4% FA for 10 minutes at room temperature, followed by another quenching step performed in 1% H_2_O_2_ (diluted in PBSTx) for 1 hour at room temperature. Following quenching, worms were rinsed and 6C8 labeling proceeded as described above.

Another modified fix was used to label planarians with anti-Acetylated Tubulin (1:1000 [clone 6-11B-1, Sigma]). Incubation times were shortened to 3 minutes in HCl for the initial kill and 2 hours at 4°C for the FA fixation. Animals were bleached and stained as described above for 5B1 antibody. After development, animals were quenched in Proteinase K and washed, blocked, and incubated in anti-Acetylated Tubulin (1:1000 [Sigma]) overnight at 4°C. The following day, worms were washed extensively and incubated overnight at 4°C in goat-anti-mouse AlexaFluor 568 (1:400 in PBSTB, [A11031, Life Technologies]) followed by additional washes.

For co-labelings with anti-SYNAPSIN (1:400 3C10, DSHB), fixations and bleaching were aqueous and had no Proteinase K treatment. SYNAPSIN labeling was always performed first using goat-anti-mouse AlexaFluor488 secondary antibodies. Following anti-SYNAPSIN labeling, samples were quenched with the Proteinase K and post-fix (as described above) before washing, blocking, and proceeding with immunostainings using the monoclonal antibodies.

Two anti-rabbit primary antibodies were used: anti-CRMP2 (1:50 [9393, Cell Signaling]) and anti-SMEDWI-1 (1:1000 [a kind gift from P. Reddien]); both were visualized with the goat-anti-rabbit Alexa Fluor 568 secondary antibodies. These were combined with the anti-mouse monoclonal primaries during the initial incubation. The secondary antibodies were also combined for incubation; development proceeded as described above.

Co-labeling experiments with Fluorescein labeled wheat germ agglutinin (WGA) or Rhodamine labeled *Lens culinaris* agglutinin (LCA) (Vector Labs) [20] were performed on intact planarians killed in N-acetyl cysteine and fixed in FA (as described in Additional file 1) with the following change: for LCA co-labeling, worms were incubated with either 6C8 or 2C4 at 1:100 dilution and subsequently labeled with goat anti-mouse AF488 at 1:500. Following secondary labeling, the worms were re-blocked for 1 hour in PBSTx with 0.6% BSA and 0.45% fish gelatin (PBSTB+FG) and incubated with WGA ([1 μg/ml [FL-1021, Vector Laboratories, Inc.]) or LCA (5 μg/ml [RL-1042, Vector Laboratories, Inc.]) and diluted in PBSTB+FG overnight at 4°C.

### Fluorescent *in situ* hybridization

Riboprobes were synthesized as previously described [19]. Animals were processed using a protocol developed for *in situ* hybridization [18]. Some modifications were made to the protocol: MABTw (Maleic Acid Buffer + 0.1% Tween-20) was used instead of TNTx for washes, and peroxidase inactivation for double-labeling was performed with 1% H_2_O_2_ in PBSTx for 1 hour instead of azide solution. After peroxidase-inactivation, animals were washed in PBSTx extensively, blocked in PBSTB for 1 hour at room temperature, and then incubated in the antibody overnight. Immunostaining proceeded as described above.

### Imaging

Single channel whole animal images were acquired with an Axiocam MRm camera mounted on a Zeiss SteREO Lumar.V12 stereomicroscope equipped with a NeoLumar S 1.5X objective running AxioVision v4.8. Higher magnification images and the whole animal image in Figure 3B were obtained on either a Zeiss Axio Observer.Z1 inverted microscope using an ApoTome for optical sectioning running AxioVision v4.8 or on a Carl Zeiss LSM710 confocal microscope running ZEN 2011 or 2012. Objectives used on the Axio Observer.Z1 were: 40 × 1.3 NA oil immersion objective, 20 × 0.8 NA objective, and 10 × 0.3 NA objective. Objectives used on the LSM710 were: 40 × 1.2 NA water immersion objective, 20 × 0.8 NA objective, or 10 × 0.3 NA objective. Images were processed in Adobe Photoshop CS4, CS6, or ImageJ 1.47f to normalize levels, color, and brightness. 3D reconstruction images were performed using ZEN 2012 software with a Z-stack containing 48 sections at 1 μm intervals.

### Cell size measurements

For cell size measurements of 2C4-labeled cells, maximum intensity projections of 20–36 × 0.54 μm slices were collected for analysis in ImageJ. The thickest diameter across the cell body was measured. A minimum of 58 cells was counted from 9 animals for each cell type analyzed.

### RNA interference

Animals were fed bacterially-expressed *gfp* or *Smed-slit* [55] double stranded RNA, as previously described [52], 8 times over a 2.5 week period. One day following the final RNAi feeding, animals were bisected along the sagittal plane and allowed to regenerate for 15 days before fixation.

## Additional files

Additional file 1:
**Detailed fixation and staining protocols.** This word document contains step-by-step instructions for the fixation/immunostaining protocols for use in the laboratory. Additional file 2: Figure S1. Smed-1H6 labels neurons found in close association with *pc2* ^+^ cell bodies (magenta). Whole-mount view of intact planarians labeled with 1H6 and processed for *in situ* hybridization to *pc2* (magenta) and counterstained with DAPI (blue). Examples of co-labeled cells are highlighted with arrowheads. Image taken to the right side of the pharynx, facing the ventral side of the worm. Anterior is to the top of the image. Images are maximum intensity projections of optical sections. Scale bar: 20 μm. Additional file 3: Figure S2. Smed-1H6 labels the subepidermal and submuscular plexuses. Single optical sections taken in the head region of intact planarians labeled with 1H6 (green) and counterstained with DAPI (blue) at 0.44 μm intervals starting on the ventral side of the animal. Depth of each optical section is displayed on each image. Dashed boxes indicate the regions of the inset images, which highlight the mesh-like pattern of 1H6 labeling at the subepidermal plexus in the 4.84-5.28 μm images and 1H6 labeling in the submuscular plexus at 11.00 μm. Anterior is to the left of the images. Scale bar: 20 μm. Additional file 4: Figure S3. Smed-2C4^+^ cells are not WGA^+^. Whole-mount view of intact planarians labeled with 2C4 (green) and wheat germ agglutinin (WGA, magenta) shows 2C4^+^ cells do not stain with WGA. (A) Image showing 2C4-S cells denoted by arrows. (B) Image of 2C4-N cells, which also lack WGA signal. Images were taken to the right side of the pharynx, facing the ventral side of the worm. Anterior is to the left. Images are maximum intensity projections. Scale bars: 50 μm. Additional file 5: Figure S4. Images of 6C8-labeled cells in close association with enteric muscular fibers. (A-B) 3-dimensional reconstruction of a Z-stack acquired from planarian enteric musculature labeled with 6G10 (magenta) and 6C8 (green) highlights the 6C8^+^ cell located outside of the enteric musculature shown in Figure 7C. Dashed boxes indicate the regions of the inset images. (A) View of 6C8-labeled cells from different angles of rotation around the X-axis (numbers in top right corner indicate the degree angle of rotation from the Y-axis). Arrows denote some examples of 6C8^+^ cells that are clearly on the luminal side of the enteric muscle boundary. (B) Images from angles of rotation around the Y-axis (numbers indicate the degree angle of rotation from the X-axis. Anterior of the animal is to the top in all images. Scale bars: 50 μm. Additional file 6: Figure S5. Smed-6C8^+^ cells are not 2C4 or LCA^+^. (A-B) Whole-mount view of intact planarians labeled with 6C8 (magenta in A, green in B), co-labeled with 2C4 (green) in A, or *Lens culinaris* agglutinin lectin (LCA, magenta) and counterstained with DAPI (blue) in B. (A) Co-staining of 6C8 and 2C4 in the primary anterior branch of the intestine demonstrates that these two antibodies label cell populations that are distinct or at different developmental stages. (B) Co-staining with LCA indicates that 6C8 does not label goblet cells in the intestine (shown in a posterior intestinal branch). The images are maximum intensity projections of optical sections acquired through the tissues described. Anterior is to the top in all images. Scale bars: 50 μm.

## Abbreviations

CMF: Calcium magnesium free media
CMF+TI: Calcium magnesium free media plus trypsin inhibitor
CNS: Central nervous system
DMEM: Dulbecco’s modified Eagle’s medium
dpa: Days post-amputation
DSHB: Developmental Studies Hybridoma Bank
DTT: Dithiothreitol
FA: Formaldehyde
FBS: Fetal bovine serum
FISH: Fluorescent *in situ* hybridization
*gpas*: G protein α-subunit
HRP: Horseradish peroxidase
LCA: *Lens culinaris* agglutinin
mAb: Monoclonal antibody
MHC: Myosin heavy chain
NP-40: Nonidet P-40
PBS: Phosphate buffered saline
PBSTB: Phosphate buffered saline plus Triton X-100 and BSA
PBSTB+FG: Phosphate buffered saline plus Triton X-100, BSA, and fish gelatin
PBSTx: Phosphate buffered saline + Triton-X 100
*pc-2*: *Prohormone convertase 2*
PNS: Peripheral nervous system
RNAi: RNA interference
SDS: Sodium dodecyl sulfate
T: Trypsin
TI: Trypsin inhibitor
VNC: Ventral nerve cords
WGA: Wheat germ agglutinin
