# Supplementary material for: Novel monoclonal antibodies to study tissue regeneration in planarians
Source: BMC Dev Biol. 2015 Jan 21;15:2. doi: 10.1186/s12861-014-0050-9 (PMC4307677; doi:10.1186/s12861-014-0050-9)
Supplement: Additional file 1: — Detailed fixation and staining protocols. This word document contains step-by-step instructions for the fixation/immunostaining protocols for use in the laboratory. [file 12861_2014_50_MOESM1_ESM.pdf]

List of Solutions:

2% HCl: diluted from 37% stock in nanowater.
PBS: 137 mM NaCl, 2.7 mM KCl, 10 mM Na_2HPO_4, 2 mM KH_2PO_4 in nanowater, pH adjusted to 7.4
PBSTx: 0.3% (v/v) Triton X-100 diluted into PBS.
4% Formaldehyde: diluted from 37% stock in PBSTx.
6% Aqueous Bleach Solution: 6% H_2O_2 dissolved from 30% stock solution in PBSTx.
5% NAC Solution: 5% N-Acetyl Cysteine (w/v) dissolved in PBS.
Proteinase-K Solution: (2 μg/ml Proteinase-K dissolved from 10 mg/ml stock, 0.1% Sodium Dodecyl Sulfate diluted from 10% (w/v) stock solution, dissolved in PBSTx.
50% Methanol: mixture of equal volumes PBSTx and 100% Methanol.
6% MeOH Bleach Solution: 6% H_2O_2 dissolved from 30% stock solution in 100% Methanol.
Reduction Solution: 50mM Dithiothreitol from 1M stock, 1% NP-40 (v/v), 0.5% Sodium Dodecyl Sulfate diluted from 10% (w/v) stock, diluted in PBS.
Blocking Solution: 1% (w/v) Bovine Serum Albumin dissolved in PBSTx.
mAb Solution: monoclonal antibody diluted Blocking Solution.
Secondary Antibody Solution: goat-anti-mouse IgG or goat-anti-mouse IgG+IgM diluted 1:1000 in Blocking Solution.
Borate Buffer: 0.1M Borate Buffer diluted from 1M Borate Buffer, pH 8.5, 0.1% Tween-20 diluted from 10% Tween-20, dissolved in nanowater.
TSA Reaction Buffer: 0.1M Borate Buffer diluted from 1M Borate Buffer, pH 8.5, 0.1% Tween-20 diluted from 10% stock Tween-20, 0.003% H_2O_2 diluted from 0.5% stock H_2O_2, 1:250 FITC-tyramide diluted 1:250, diluted in nanowater.
DAPI Solution: 0.05 μg/mL 4',6-diamidino-2-phenylindole (DAPI) diluted from 10 mg/mL stock in PBSTx.
Tyramide-PBSTI Solution: 10mM Imidazole, FITC-tyramide diluted 1:250-1:1000 in PBSTx.
Tyramide-PBSTI Solution+H_2O_2: 10mM Imidazole, 1:1000 FITC-tyramide, and 0.015% H_2O_2 diluted from 30% stock, diluted in PBSTx.

Protocol for Fixation "HCl-FA"

<u>Day 1</u>(kill, fix, bleach)

1) Prepare ice-bath and pre-chill **2% HCl**.
2) Transfer worms (length 2-6 mm) in planarian water to appropriate vial for fixation: (1.5 ml conical for up to 10 worms, small scintillation vial for up to 50 small planarians, large scintillation vial for up to 100 small planarians or 15 ml conical for 10 to 100 planarians).
3) Replace planarian water with **2% HCl**, incubate for 5 minutes, alternating 1 minute on ice, 1 minute inverting.
4) Rinse once with **PBS**.
5) Replace with **4% Formaldehyde**, incubate for 15 minutes at RT, rocking.
6) Remove **4% Formaldehyde** and rinse worms twice with **PBSTx**.
7) Replace **PBSTx** with **6% Aqueous Bleach Solution**, leave under direct light overnight at RT.

<u>Day 2</u> (rinse, proceed to blocking)

8) Remove **6% Aqueous Bleach Solution** and rinse specimens twice with **PBSTx**.
9) Proceed to Blocking/Staining.

Protocol for Fixation "HCl-FA-Cold"

<u>Day 1</u>(kill, long fix, bleach)

1) Prepare ice-bath and pre-chill **2% HCl**.
2) Transfer worms (length 2-6 mm) in planarian water to appropriate vial for fixation: (1.5 ml conical for up to 10 worms, small scintillation vial for up to 50 small planarians, large scintillation vial for up to 100 small planarians or 15 ml conical for 10 to 100 planarians).
3) Replace planarian water with **2% HCl**, incubate for 5 minutes, alternating 1 minute on ice, 1 minute inverting.
4) Rinse once with **PBS**.
5) Replace with pre-chilled **4% Formaldehyde**, incubate for 6 hours at 4°C, rocking.
6) Remove **4% Formaldehyde** and rinse worms twice with **PBSTx**.
7) Replace **PBSTx** with **6% Aqueous Bleach Solution**, leave under direct light overnight at RT.

<u>Day 2</u> (rinse, proceed to blocking)

8) Remove **6% Aqueous Bleach Solution** and rinse specimens twice with **PBSTx**.
9) Proceed to Blocking/Staining.

Protocol for Fixation "NAC-FA"

Day 1(kill, fix, bleach)

1) Transfer worms (length 2-6 mm) in planarian water to appropriate vial for fixation: (1.5 ml conical for up to 10 worms, small scintillation vial for up to 50 small planarians, large scintillation vial for up to 100 small planarians or 15 ml conical for 10 to 100 planarians).
2) Replace planarian water with **5% NAC Solution**, incubate for 5 minutes at RT, rocking.
3) Rinse once with **PBS**.
4) Replace with **4% Formaldehyde**, incubate for 15 minutes at RT, rocking.
5) Remove **4% Formaldehyde** and rinse worms twice with **PBSTx**.
6) Replace **PBSTx** with **6% Aqueous Bleach Solution**, leave under direct light overnight at RT.

Day 2 (rinse, proceed to block)

7) Remove **6% Aqueous Bleach Solution** and rinse specimens twice with **PBSTx**.
8) Proceed to Blocking/Staining.

Protocol for Fixation "HCl-FA-ProtK"

Day 1(kill, fix, bleach)

1) Prepare ice-bath and pre-chill **2% HCl**.
2) Transfer worms (length 2-6 mm) in planarian water to appropriate vial for fixation: (1.5 ml conical for up to 10 worms, small scintillation vial for up to 50 small planarians, large scintillation vial for up to 100 small planarians or 15 ml conical for 10 to 100 planarians).
3) Replace planarian water with **2% HCl**, incubate for 5 minutes, alternating 1 minute on ice, 1 minute inverting.
4) Rinse once with **PBS**.
5) Replace with **4% Formaldehyde**, incubate for 15 minutes at RT, rocking.
6) Remove **4% Formaldehyde** and rinse worms twice with **PBSTx**.
7) Replace **PBSTx** with **6% Aqueous Bleach Solution**, leave under direct light overnight at RT.

Day 2 (Proteinase-K, post-fix, proceed to block)

8) Remove **6% Aqueous Bleach Solution** and rinse specimens twice with **PBSTx**.
9) Add **Proteinase-K Solution**, incubate for 10 minutes at RT, rocking.
10) Replace **Proteinase-K Solution** with **4% Formaldehyde**, incubate for 10 minutes at RT, rocking.
11) Remove **4% Formaldehyde** and rinse worms twice with **PBSTx**.
12) Proceed to Blocking/Staining.

Protocol for Fixation "NAC-FA-Me"

Day 1 (kill, fix, dehydrate, bleach)

1) Transfer worms (length 2-6 mm) in planarian water to appropriate vial for fixation: (1.5 ml conical for up to 10 worms, small scintillation vial for up to 50 small planarians, large scintillation vial for up to 100 small planarians or 15 ml conical for 10 to 100 planarians).
2) Replace planarian water with **5% NAC Solution**, incubate for 5 minutes at RT, rocking.
3) Rinse once with **PBS**.
4) Replace with **4% Formaldehyde**, incubate for 15 minutes at RT, rocking.
5) Remove **4% Formaldehyde** and rinse worms twice with **PBSTx**.
6) Add **50% Methanol Solution**, incubate for 5-10 minutes at RT, rocking.
7) Replace **50% Methanol Solution** with 100% Methanol, incubate for 5-10 minutes at RT (rocking), and then transfer to -20°C for ≥ 1 hour.
8) Replace Methanol with **6% MeOH Bleach Solution**, leave under direct light overnight at RT.

Day 2 (rehydrate, proceed to block)

9) Remove **6% MeOH Bleach Solution** and rinse specimens twice with 100% Methanol
   Note: can store specimens at -20°C for up to 1 month.
10) Add **50% Methanol Solution**, incubate for 5-10 minutes at RT, rocking.
11) Replace **50% Methanol Solution** with **PBSTx**, incubate for 5-10 minutes at RT, rocking.
12) Rinse twice with **PBSTx**.
13) Proceed to Blocking/Staining.

Protocol for Fixation "NAC-FA-Me-ProtK"

Day 1 (kill, fix, dehydrate, bleach)

1) Transfer worms (length 2-6 mm) in planarian water to appropriate vial for fixation: (1.5 ml conical for up to 10 worms, small scintillation vial for up to 50 small planarians, large scintillation vial for up to 100 small planarians or 15 ml conical for 10 to 100 planarians).
2) Replace planarian water with **5% NAC Solution**, incubate for 5 minutes at RT, rocking.
3) Rinse once with **PBS**.
4) Replace with **4% Formaldehyde**, incubate for 15 minutes at RT, rocking.
5) Remove **4% Formaldehyde** and rinse worms twice with **PBSTx**.
6) Add **50% Methanol Solution**, incubate for 5-10 minutes at RT, rocking.
7) Replace **50% Methanol Solution** with 100% Methanol, incubate for 5-10 minutes at RT (rocking), and then transfer to -20°C for ≥ 1 hour.
8) Replace Methanol with **6% MeOH Bleach Solution**, leave under direct light overnight at RT.

Day 2 (rehydrate, Proteinase-K, post-fix, proceed to block)

9) Remove **6% MeOH Bleach Solution** and rinse specimens twice with 100% Methanol
   Note: can store specimens at -20°C for up to 1 month.
10) Add **50% Methanol Solution**, incubate for 5-10 minutes at RT, rocking.
11) Replace **50% Methanol Solution** with **PBSTx**, incubate for 5-10 minutes at RT, rocking.
12) Replace **PBSTx** with **Proteinase-K Solution**, incubate for 10 minutes at RT, rocking.
13) Replace **Proteinase-K Solution** with **4% Formaldehyde**, incubate for 10 minutes at RT, rocking.
14) Remove **4% Formaldehyde** and rinse worms twice with **PBSTx**.
15) Proceed to Blocking/Staining.

Protocol for Fixation “NAC-FA-Red-Me-ProtK”

Day 1 (kill, fix, reduce/permeabilize, dehydrate, bleach)

1) Transfer worms (length 2-6 mm) in planarian water to appropriate vial for fixation: (1.5 ml conical for up to 10 worms, small scintillation vial for up to 50 small planarians, large scintillation vial for up to 100 small planarians or 15 ml conical for 10 to 100 planarians).
2) Replace planarian water with **5% NAC Solution**, incubate for 5 minutes at RT, rocking.
3) Rinse once with **PBS**.
4) Replace with **4% Formaldehyde**, incubate for 15 minutes at RT, rocking.
5) Remove **4% Formaldehyde** and rinse worms twice with **PBSTx**.
6) Add preheated (to 37°C in waterbath) **Reduction Solution**, incubate for 5-10 minutes at 37°C (in water bath) with gentle, intermittent inversions of the tube by hand.
7) Rince once with **PBSTx**.
8) Add **50% Methanol Solution**, incubate for 5-10 minutes at RT, rocking.
9) Replace **50% Methanol Solution** with 100% Methanol, incubate for 5-10 minutes at RT (rocking), and then transfer to -20°C for ≥ 1 hour.
10) Replace Methanol with **6% MeOH Bleach Solution**, leave under direct light overnight at RT.

Day 2 (rehydrate, Proteinase-K, post-fix, proceed to block)

11) Remove **6% MeOH Bleach Solution** and rinse specimens twice with 100% Methanol
    Note: can store specimens at -20°C for up to 1 month.
12) Add **50% Methanol Solution**, incubate for 5-10 minutes at RT, rocking.
13) Replace **50% Methanol Solution** with **PBSTx**, incubate for 5-10 minutes at RT, rocking.
14) Replace **PBSTx** with **Proteinase-K Solution**, incubate for 10 minutes at RT, rocking.
15) Replace **Proteinase-K Solution** with **4% Formaldehyde**, incubate for 10 minutes at RT, rocking.
16) Remove **4% Formaldehyde** and rinse worms twice with **PBSTx**.
17) Proceed to Blocking/Staining.

Protocol for Fixation "HCl-FA-Me"

<u>Day 1</u>(kill, fix, dehydrate, bleach)

1) Prepare ice-bath and pre-chill **2% HCl**.
2) Transfer worms (length 2-6 mm) in planarian water to appropriate vial for fixation: (1.5 ml conical for up to 10 worms, small scintillation vial for up to 50 small planarians, large scintillation vial for up to 100 small planarians or 15 ml conical for 10 to 100 planarians).
3) Replace planarian water with **2% HCl**, incubate for 5 minutes, alternating 1 minute on ice, 1 minute inverting.
4) Rinse once with **PBS**.
5) Replace with **4% Formaldehyde**, incubate for 15 minutes at RT, rocking.
6) Remove **4% Formaldehyde** and rinse worms twice with **PBSTx**.
7) Add **50% Methanol Solution**, incubate for 5-10 minutes at RT, rocking.
8) Replace **50% Methanol Solution** with 100% Methanol, incubate for 5-10 minutes at RT (rocking), and then transfer to -20°C for ≥ 1 hour.
9) Replace Methanol with **6% MeOH Bleach Solution**, leave under direct light overnight at RT.

<u>Day 2</u> (rehydrate, proceed to block)

10) Remove **6% MeOH Bleach Solution** and rinse specimens twice with 100% Methanol
    Note: can store specimens at -20°C for up to 1 month.
11) Add **50% Methanol Solution**, incubate for 5-10 minutes at RT, rocking.
12) Replace **50% Methanol Solution** with **PBSTx**, incubate for 5-10 minutes at RT, rocking.
13) Rinse twice with **PBSTx**.
14) Proceed to Blocking/Staining.

Blocking / Staining:

1) Move worms to wells in 24, 48, or 96 well-plate in **PBSTx**.
2) Replace **PBSTx** with **Blocking Solution**, incubate 4 hours at RT, rocking.
3) Replace **Blocking Solution** with **mAb Solution**, incubate overnight at 4°C, rocking.
4) Remove **mAb Solution** (Note: **mAb Solution** can be reserved at 4°C and reused [add NaN_3 to a final concentration of 0.02% w/v]). Rinse three times with **PBSTx**, and then wash 6-8 hours, replacing **PBSTx** at least six times.
5) Add **Blocking Solution** and incubate for 1 hour at RT, rocking.
6) Replace **Blocking Solution** with **Secondary Antibody Solution**. Incubate overnight at 4°C, rocking.
7) Remove **Secondary Antibody Solution**. Rinse three times with **PBSTx**, and then wash 6-8 hours, replacing **PBSTx** at least six times. Proceed to development.

TSA developing with Borate Buffer

1) Incubate animals for 5 minutes in **Borate Buffer** at RT, rocking.
2) Note: keep plate wrapped in foil to protect from light from this point forward. Incubate animals for 10 minutes in **TSA Reaction Buffer** at RT, rocking.
3) Spike in additional H_20_2 (1.5 ul of 0.5% H_20_2 per 250 ul of TSA Reaction Buffer) and incubate for an additional 10 minutes at RT, rocking.
4) Wash three times in **PBSTx** (incubating each wash step at RT for 10 minutes, rocking), then continue to wash in **PBSTx** overnight at 4°C (continue washing next day at RT in **PBSTx**).
5) Incubate overnight in DAPI (0.05 μg/mL diluted in **PBSTx**.)

TSA developing with PBSTI

1) Note: keep plate wrapped in foil to protect from light from this point forward. Incubate animals for 30 minutes in **Tyramide-PBSTI Solution**.
2) Incubate animals for 5 minutes in **Tyramide-PBSTI Solution+H_2O_2**.
3) Wash three times in **PBSTx** (incubating each wash step at RT for 10 minutes, rocking), then continue to wash in **PBSTx** overnight at 4°C (continue washing next day at RT in **PBSTx**).
4) Incubate overnight in DAPI (0.05 μg/mL diluted in **PBSTx**.)
