# Supplementary figures and images for: Novel monoclonal antibodies to study tissue regeneration in planarians
Source: BMC Dev Biol. 2015 Jan 21;15:2. doi: 10.1186/s12861-014-0050-9 (PMC4307677; doi:10.1186/s12861-014-0050-9)

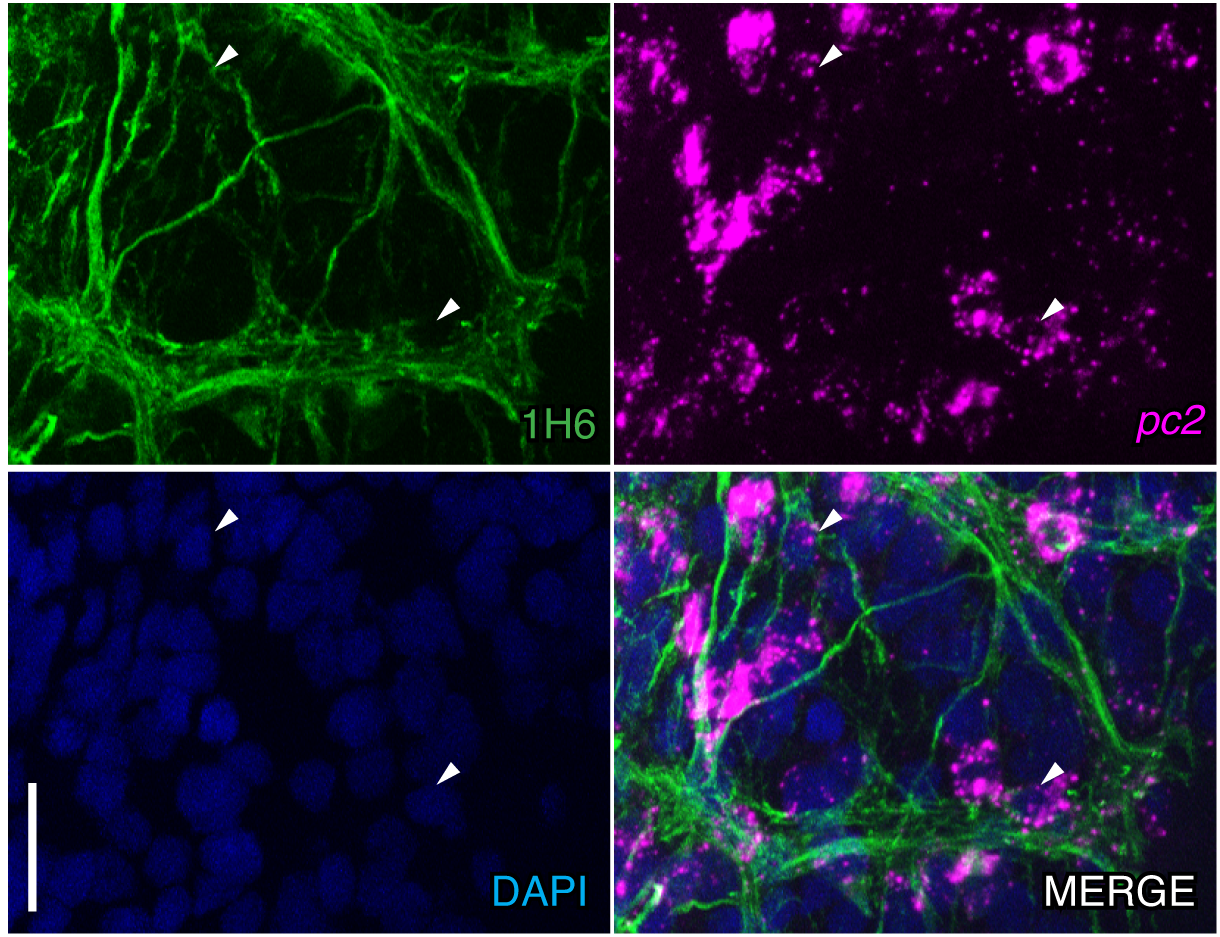

Supplement: Additional file 2: Figure S1. — Smed-1H6 labels neurons found in close association with pc2 + cell bodies (magenta). Whole-mount view of intact planarians labeled with 1H6 and processed for in situ hybridization to pc2 (magenta) and counterstained with DAPI (blue). Examples of co-labeled cells are highlighted with arrowheads. Image taken to the right side of the pharynx, facing the ventral side of the worm. Anterior is to the top of the image. Images are maximum intensity projections of optical sections. Scale bar: 20 μm. [file 12861_2014_50_MOESM2_ESM.tiff]

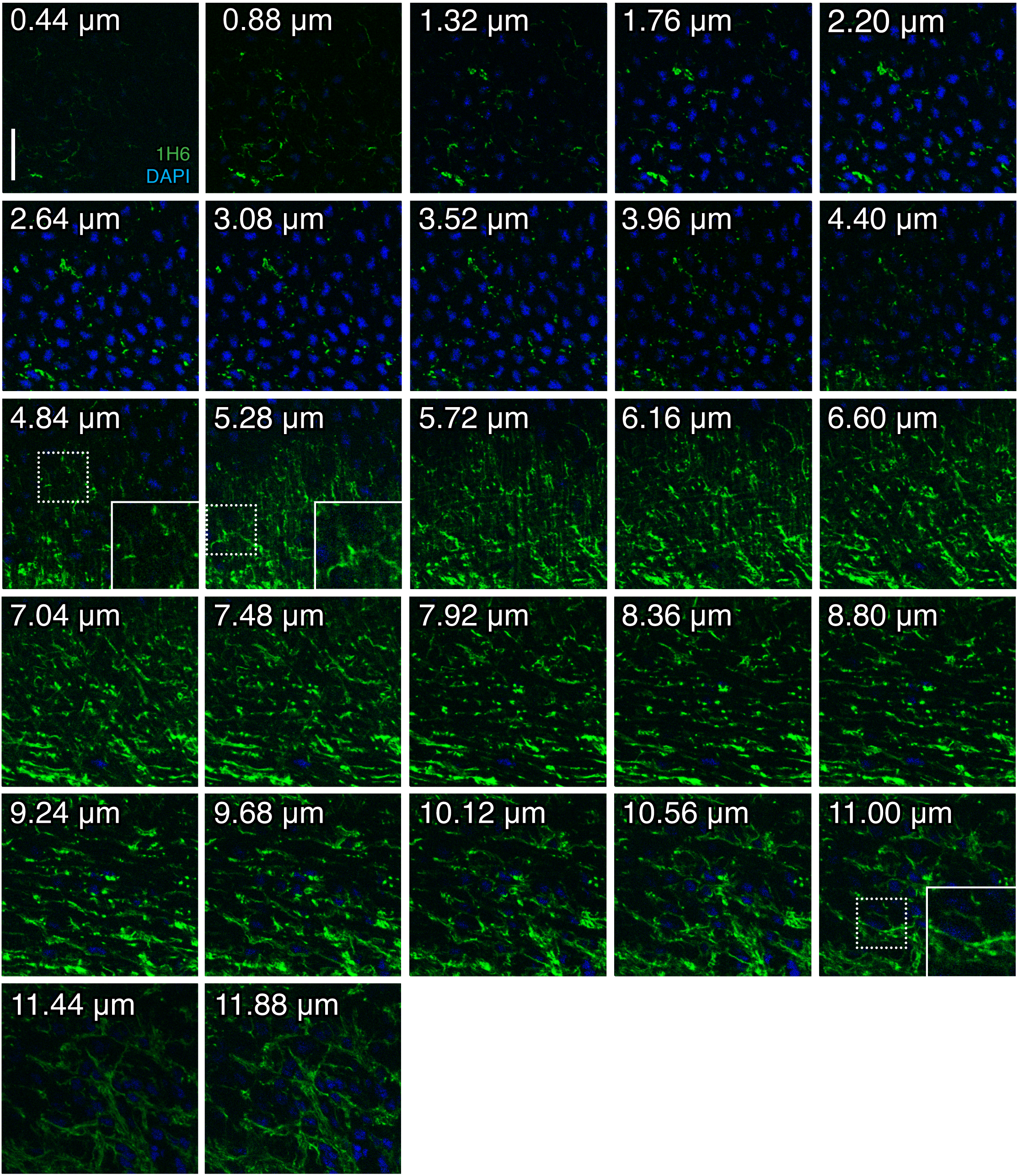

Supplement: Additional file 3: Figure S2. — Smed-1H6 labels the subepidermal and submuscular plexuses. Single optical sections taken in the head region of intact planarians labeled with 1H6 (green) and counterstained with DAPI (blue) at 0.44 μm intervals starting on the ventral side of the animal. Depth of each optical section is displayed on each image. Dashed boxes indicate the regions of the inset images, which highlight the mesh-like pattern of 1H6 labeling at the subepidermal plexus in the 4.84-5.28 μm images and 1H6 labeling in the submuscular plexus at 11.00 μm. Anterior is to the left of the images. Scale bar: 20 μm. [file 12861_2014_50_MOESM3_ESM.tiff]

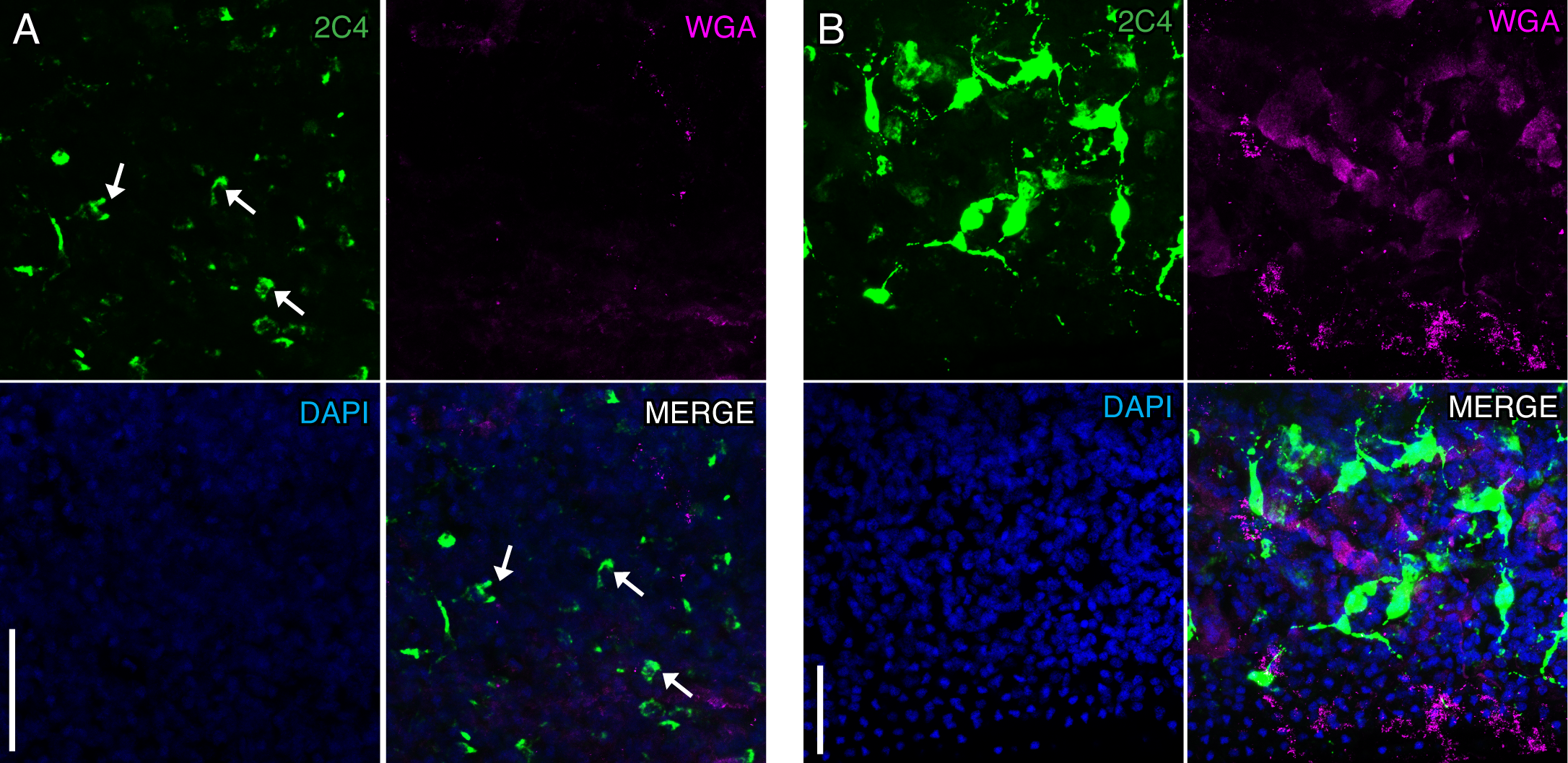

Supplement: Additional file 4: Figure S3. — Smed-2C4+ cells are not WGA+. Whole-mount view of intact planarians labeled with 2C4 (green) and wheat germ agglutinin (WGA, magenta) shows 2C4+ cells do not stain with WGA. (A) Image showing 2C4-S cells denoted by arrows. (B) Image of 2C4-N cells, which also lack WGA signal. Images were taken to the right side of the pharynx, facing the ventral side of the worm. Anterior is to the left. Images are maximum intensity projections. Scale bars: 50 μm. [file 12861_2014_50_MOESM4_ESM.tiff]

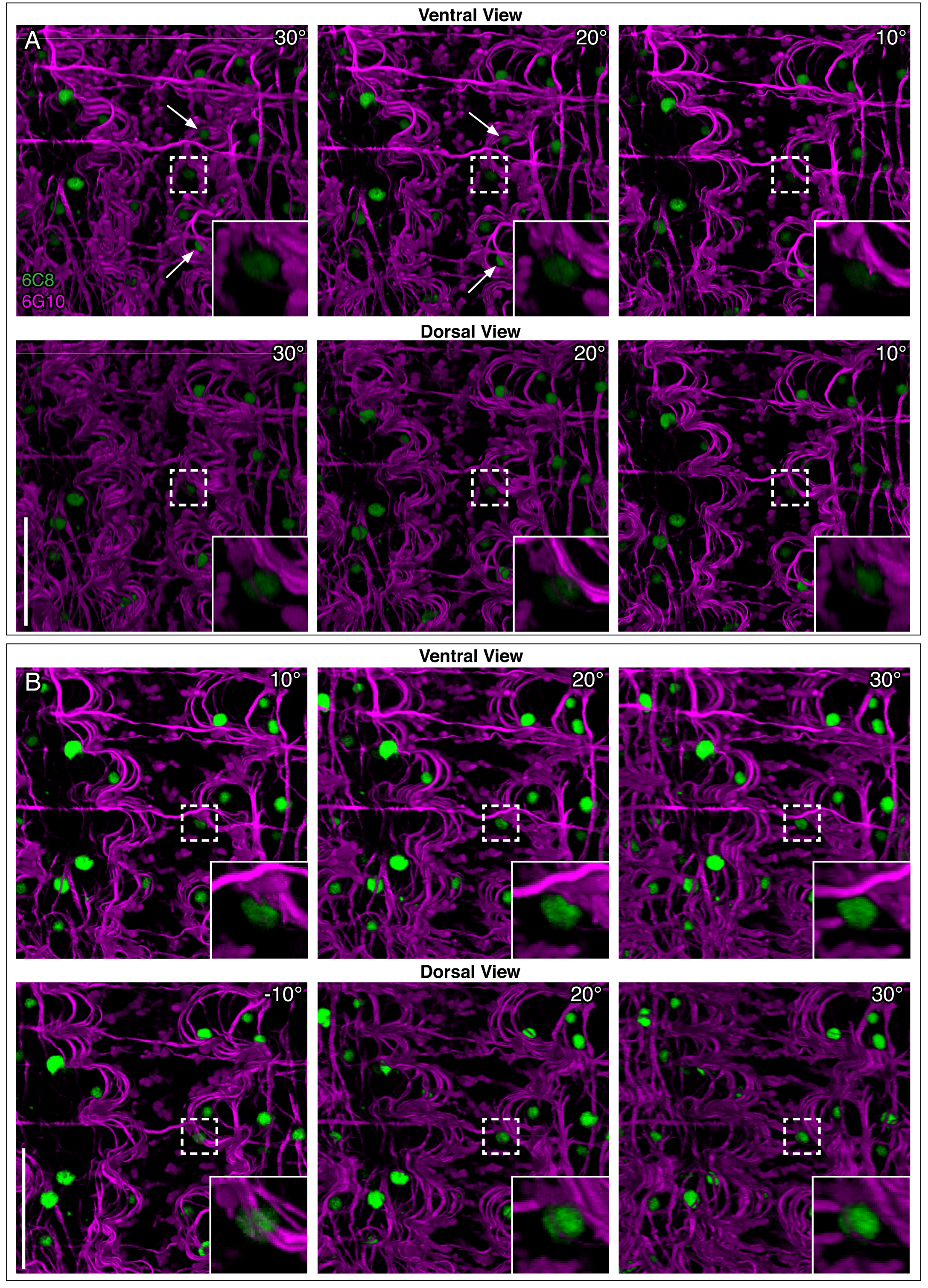

Supplement: Additional file 5: Figure S4. — Images of 6C8-labeled cells in close association with enteric muscular fibers. (A-B) 3-dimensional reconstruction of a Z-stack acquired from planarian enteric musculature labeled with 6G10 (magenta) and 6C8 (green) highlights the 6C8+ cell located outside of the enteric musculature shown in Figure 7C. Dashed boxes indicate the regions of the inset images. (A) View of 6C8-labeled cells from different angles of rotation around the X-axis (numbers in top right corner indicate the degree angle of rotation from the Y-axis). Arrows denote some examples of 6C8+ cells that are clearly on the luminal side of the enteric muscle boundary. (B) Images from angles of rotation around the Y-axis (numbers indicate the degree angle of rotation from the X-axis. Anterior of the animal is to the top in all images. Scale bars: 50 μm. [file 12861_2014_50_MOESM5_ESM.tiff]

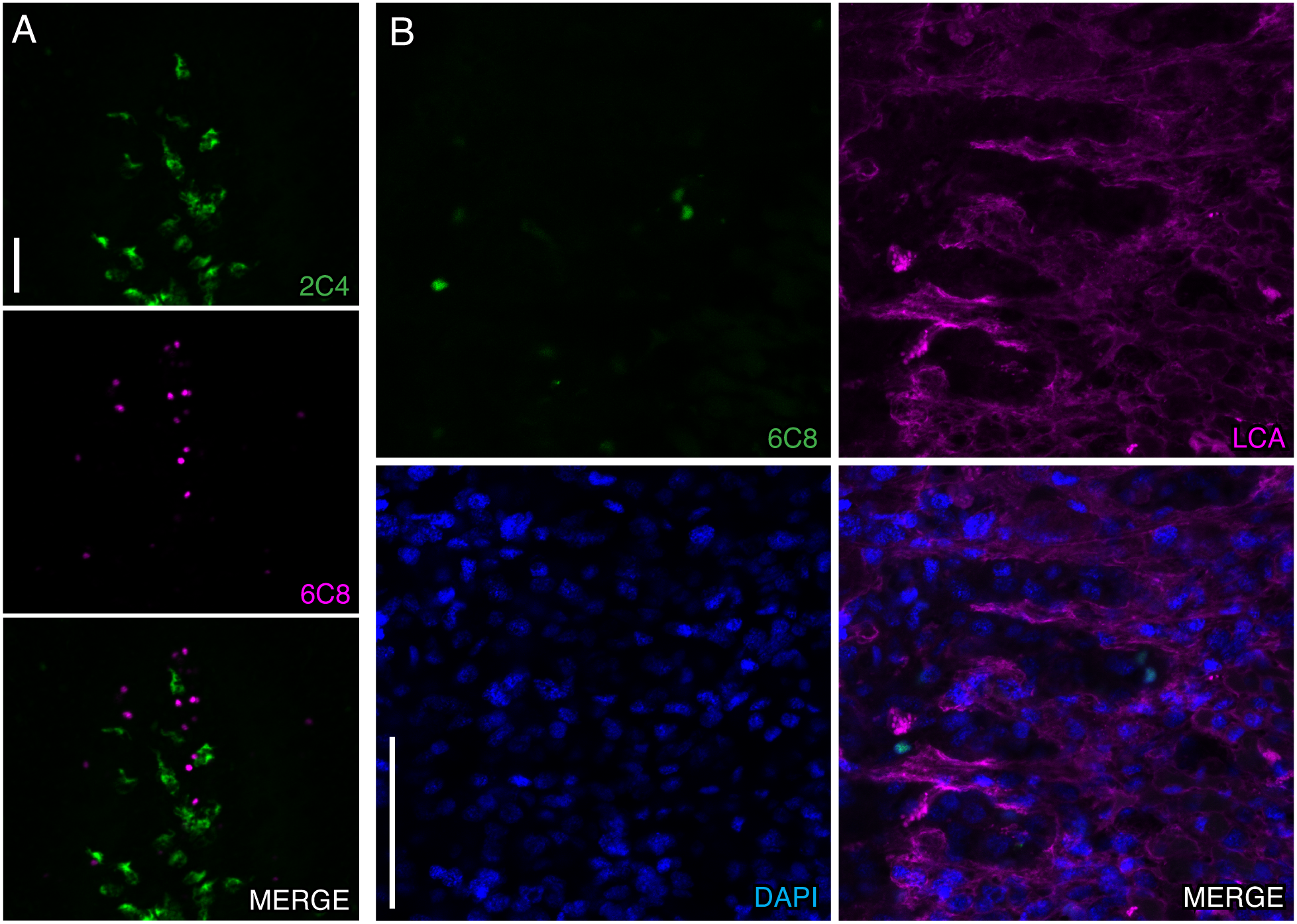

Supplement: Additional file 6: Figure S5. — Smed-6C8+ cells are not 2C4 or LCA+. (A-B) Whole-mount view of intact planarians labeled with 6C8 (magenta in A, green in B), co-labeled with 2C4 (green) in A, or Lens culinaris agglutinin lectin (LCA, magenta) and counterstained with DAPI (blue) in B. (A) Co-staining of 6C8 and 2C4 in the primary anterior branch of the intestine demonstrates that these two antibodies label cell populations that are distinct or at different developmental stages. (B) Co-staining with LCA indicates that 6C8 does not label goblet cells in the intestine (shown in a posterior intestinal branch). The images are maximum intensity projections of optical sections acquired through the tissues described. Anterior is to the top in all images. Scale bars: 50 μm. [file 12861_2014_50_MOESM6_ESM.tiff]
